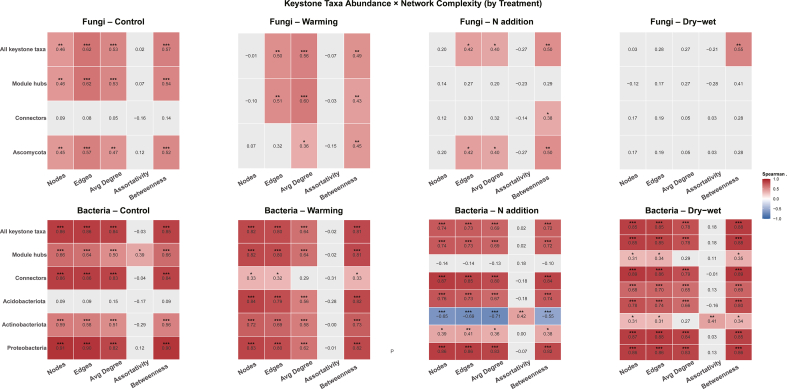# Divergent responses of microbial networks to global change scenarios during alpine cushion plant degradation

**DOI:** 10.1016/j.pld.2026.04.010

**Published:** 2026-05-04

**Authors:** Mengdie Yin, Yaojun Ye, Xin Liu, Wanyin Xiong, Shijian Liu, Hang Sun, Wenguang Sun, Yazhou Zhang

**Affiliations:** a School of Life Sciences, Yunnan Normal University, Kunming 650000, China; b State Key Laboratory of Plant Diversity and Specialty Crops/Yunnan Key Laboratory for Plant Diversity and Biogeography, Kunming Institute of Botany, Chinese Academy of Sciences, Kunming 650201, China; c School of Ecology and Environment, Southwest Forestry University, Kunming 650224, China; d Southwest United Graduate School, Kunming 650092, China

**Keywords:** Alpine tundra, Cushion plant degradation, Soil microcosm, Global change scenarios, Microbial co-occurrence networks

## Abstract

The degradation of alpine cushion plants is known to disrupt soil microbial networks. To explore the mechanisms behind this, we collected soils across a degradation sequence on the Qinghai-Xizang Plateau and, using a microcosm approach, simulated the effects of three global change factors: warming (T), nitrogen addition (N), and dry-wet cycling (D). Our findings indicated that fungal and bacterial networks exhibited contrasting structures and response patterns. Fungal networks displayed hub-dependent structure: they maintained complexity and enhanced robustness under T, N, and D, yet showed vulnerability when keystone nodes were preferentially removed. Bacterial networks exhibited connector-mediated redundancy that conferred high baseline robustness but limited adaptive capacity; their robustness did not increase under T or N, though assortativity rose under N. Network complexity showed nonlinear shifts across degradation stages. At two thresholds—individual-level Stage 3–4 and community-level balanced-to-stable transition—fungal networks reorganized while bacterial networks simplified. Both abiotic and biotic factors predicted network dynamics. Abiotic predictors included microbial biomass, nutrients, pH, and polyphenol oxidase (PPO) activity. Beyond these, keystone taxa abundance emerged as a biotic driver strongly correlated with bacterial network complexity, though this relationship was attenuated under warming. We propose a management framework that prioritizes protecting bacterial network integrity through mitigating warming and nitrogen deposition, targets pre-threshold stages as intervention windows, and integrates soil-microbial indicators for early warning. This study provides a practical basis for predicting and managing microbial network dynamics in alpine tundra under global change.

## Highlights

• Fungal networks remained stable; bacterial networks were vulnerable.
• Nonlinear thresholds occurred at key degradation stages.
• Initial soil properties predicted microbial responses.
• A vulnerability-focused framework is proposed for ecosystem management.

## 1 Introduction

Alpine tundra and subnival ecosystems form the highest elevation vegetation belts on land. They support biological communities uniquely adapted to periglacial environments with persistent low temperatures, intense solar radiation, and short growing seasons (Körner, 2021; Sun et al., 2014; Yang et al., 2025; Zhang et al., 2021). These ecosystems are exceptionally sensitive to climate change, more so than lowland systems, making them crucial sentinels for monitoring global environmental shifts (Beniston, 2003; Pepin et al., 2015). Within these fragile zones, ecosystem degradation primarily involves vegetation transition from alpine tundra to grassland, a process often initiated by the degradation of cushion plants (Chen et al., 2024). As foundation species and ecosystem engineers, cushion plants are fundamental to alpine tundra functioning (Zhang et al., 2022). Their three-dimensional structures buffer microclimatic extremes, enhance soil moisture retention and nutrient conservation, and generate specialized sub-communities for invertebrates, plants, and microorganisms. These roles collectively enhance aboveground and belowground biodiversity and ecosystem multifunctionality at both community and individual levels (Chen et al., 2024; Zhang et al., 2025c). Consequently, the degradation of these keystone plants leads to fundamental deterioration of core ecosystem processes. Current research, however, remains primarily focused on aboveground ecological dynamics, particularly shifts in plant and arthropod diversity. This focus has resulted in a significant knowledge gap regarding how belowground biodiversity responds to degradation, especially the complex interactions within soil microbial communities (Chen et al., 2015, 2021; Schöb et al., 2013). Moreover, it is unclear whether these belowground responses are consistent across different microbial taxa and global change scenarios, or whether they vary depending on the cushion plant degradation stage.

Soil microbial communities are fundamental components in maintaining ecosystem function and stability in alpine regions (Praeg et al., 2025). Microbial networks reflect potential interspecies associations. Their complexity (e.g., the number of nodes and edges) and robustness are two key dimensions for quantifying such networks, which jointly respond to environmental changes and are closely linked to ecosystem function (Luo et al., 2023, Yuan et al., 2021). However, these responses are highly scenario/climate-dependent. For example, nitrogen deposition reduces network complexity in subtropical forests but enhances it in alpine meadows (Liu et al., 2025; Tian et al., 2017). Similarly, long-term warming can increase network robustness in grassland ecosystems but may weaken it in forest ecosystems (Munday et al., 2025; Yuan et al., 2021). Importantly, current understanding predominantly derives from low-elevation ecosystems. Microbial communities in alpine tundra, shaped by prolonged adaptation to extreme conditions, may exhibit fundamentally different response patterns (Liu et al., 2024; Schmidt et al., 2009). Consequently, empirical data from such systems are lacking, making it difficult to predict how global change will reshape their microbial network dynamics.

These scenario-dependent responses arise, in part, from inherent differences in the functional traits and life-history strategies of microbial taxa (Powell et al., 2015a; Zhang et al., 2025a, 2025b). For example, in Arctic tundra warming experiments, bacterial diversity declined significantly while fungal diversity remained stable (Mackelprang et al., 2011). This divergence may be attributed to fungi's ability to expand spatially via mycelial networks to utilize diverse substrates through extracellular enzyme production (Allison et al., 2013; Peay et al., 2016). Similarly, under nitrogen deposition, long-term fertilization experiments demonstrate that bacterial network connectivity often decreases more sharply than fungal connectivity (Powell et al., 2015b). Taxon-specific responses are also evident during moisture fluctuations, where fungi often show greater resilience due to hydrophobic mycelial networks that enhance water acquisition, contrasting with bacterial dependence on aqueous diffusion (de Vries et al., 2018). Therefore, predicting microbial responses to global change requires taxon-specific frameworks (Zhang et al., 2024). This is particularly critical for understudied systems like the alpine tundra, where such responses remain largely unknown and may govern ecosystem outcomes.

Vegetation degradation invariably alters soil properties, such as pH and nutrient availability (Delgado-Baquerizo et al., 2013). These altered soil properties act as environmental filters, reconstituting microbial interactions and leading to a substantial reorganization of microbial networks across degradation stages (Karimi et al., 2018; Wardle et al., 2004). For example, in alpine tundra of the Qinghai-Xizang Plateau, microbial network complexity shows a nonlinear trajectory: it declines during moderate degradation but may partially recover in late stages following pioneer plant colonization (Zhang et al., 2025c, 2025d). Such stage-dependent patterns suggest that microbial responses are governed by key soil properties (Allison and Martiny, 2008). Thus, the path of network reorganization may be profoundly influenced by where the system begins—that is, the initial properties of the soil. This aligns with the concept of soil legacy effects, wherein initial properties create a persistent template that channels subsequent microbial community assembly and network dynamics through ecological feedbacks (Philippot et al., 2024). Consequently, investigating the relationship between initial soil properties and microbial network metrics is central to understanding and predicting the trajectory of ecosystem degradation in alpine tundra.

The alpine tundra of the Qinghai-Xizang Plateau is a critically vulnerable and functionally pivotal ecosystem that harbors one of the planet's most diverse assemblages of cushion plants (Sun et al., 2025; Yang et al., 2025; Zhang et al., 2022). As foundational ecosystem engineers, these plants constitute essential elements of alpine pioneer communities. Their degradation, therefore, signifies the loss of fundamental structural and functional units that sustain the entire ecosystem (Chen et al., 2024). This loss can have cascading effects on the underlying soil microbial networks essential for ecosystem functioning (Zhang et al., 2025c). Consequently, different stages of degradation create distinct environmental contexts that integrate the impacts of global change scenarios, altered soil properties, and the specific strategies of microbial taxa. This integration likely explains the divergent, nonlinear dynamics of microbial network reorganization during degradation. However, a systematic understanding of how these degradation stages shape taxa-specific microbial network responses in alpine tundra remains lacking.

Focusing on the alpine tundra of the eastern Qinghai-Xizang Plateau—a degraded cushion plant system transitioning to grassland—this study investigates how microbial network complexity and robustness respond to simulated global change scenarios (warming, nitrogen addition, and dry-wet cycling) across different cushion plant degradation stages. Combining in situ gradient sampling with laboratory microcosm experiments (Fig. 1), this research aims to: (1) discriminate the individual effects of warming, nitrogen addition, and dry-wet cycling on microbial network complexity and robustness; (2) identify key thresholds in network complexity along degradation stages; (3) compare response patterns between bacterial and fungal communities; and (4) determine how initial soil properties and biotic factors (e.g., keystone taxa abundance) predict network responses. Our investigation addresses three fundamental questions: (1) How do global change scenarios—warming, nitrogen addition, and dry-wet cycling—respectively influence soil microbial networks in degraded alpine tundra? (2) How do network responses vary with degradation stages and between microbial taxa? (3) Which soil abiotic or biotic factors most effectively predict microbial network responses to global change? Through multiscale analysis of microbial network, this research provides practical insights into alpine tundra adaptation to global change while establishing theoretical foundations for ecology early-warning systems utilizing microbial indicators. These findings will inform conservation strategies for vulnerable high-elevation ecosystems.

**Fig. 1:**
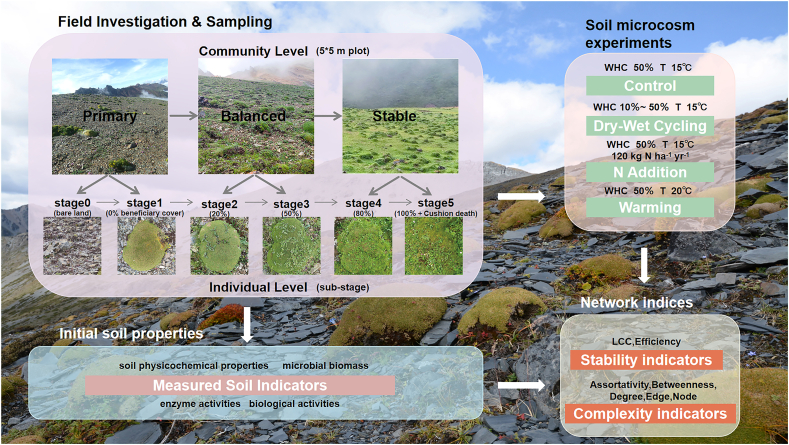
**Experimental workflow for evaluating microbial network responses to global change scenarios across cushion plant degradation stages in the alpine tundra.** A nested, multi-scale sampling strategy (based on a space-for-time substitution method) was employed: soil samples were collected from three community degradation stages (Primary, Balanced, Stable) and six individual degradation substages (defined by beneficiary species coverage). After characterizing initial soil properties, soils were exposed to four treatments (three global change scenarios and a control) in a microcosm experiment. Microbial co-occurrence networks were subsequently constructed to assess network complexity and robustness. Abbreviations: WHC, Water-Holding Capacity; LCC, Largest Connected Component. The photograph of the primary stage was taken by Jianguo Chen, while all other photographs were taken by Yazhou Zhang.

## 2 Materials and methods

### 2.1 Study site and experimental design

The study was conducted in the alpine tundra/subnival ecosystem of Baima Snow Mountain, situated on the eastern margin of the Qinghai-Xizang Plateau (28°28′29.61″N, 98°59′54.68″E). This area is a well-defined transitional ecotone between alpine meadow and tundra zones. The regional climate is a severe alpine cold-desert type, predominantly influenced by the summer monsoon that delivers most of the annual precipitation during the growing season (Wang, 2006; Zhang, 1997). Mean annual temperature ranges from—1.5 to 2.5 °C. The growing season is brief, extending from mid-May to late September, during which the soil temperature at 10 cm depth exhibits pronounced diurnal fluctuation, typically between 5 and 15 °C. The dominant foundation species across this landscape is the cushion plant *Arenaria polytrichoides* (Zhang et al., 2025c).

To investigate alpine tundra ecosystem degradation, we adopted a chronosequence framework, using a “space-for-time” substitution approach based on field monitoring initiated in 2010 (Chen et al., 2024; Yang et al., 2010). This approach was also supported by clear shifts in key initial soil properties across degradation stages (Table S1–S2). At the individual level, SOC increased from 12.1 ± 3.3 g kg^−1^ at Stage 0–131.7 ± 23.0 g kg^−1^ at Stage 4 and then declined to 105.2 ± 48.8 g kg^−1^ at Stage 5, while TN showed the same overall pattern (0.15 ± 0.03%, 1.13 ± 0.18%, and 0.98 ± 0.40%, respectively). Soil pH showed a non-monotonic pattern, reaching its lowest value at Stage 2 (5.35 ± 0.09). At the community level, SOC and TN were highest in the Stable community (SOC: 142.6 ± 50.3 g kg^−1^; TN: 1.32 ± 0.38%), whereas pH remained relatively stable across the Primary, Balanced, and Stable communities (5.84 ± 0.17, 5.68 ± 0.15, and 5.82 ± 0.21, respectively). These data provide the baseline context for the subsequent analyses of initial soil properties mediating network responses.

Using this framework, we captured the degradation trajectory of cushion plant communities at two integrated spatial levels. At the broader community level, long-term observations have delineated three sequential degradation stages along a vegetation transition gradient from alpine tundra to grassland (Fig. 1): **(1) Primary Community (Cushion-plant dominated):** Cushion plants dominate, acting as ecosystem engineers that ameliorate extreme abiotic conditions and facilitate the establishment of beneficiary plant species. **(2) Balanced Community (Co-existence):** A phase of species co-existence where beneficiary plants increasingly invade the cushion canopy, introducing competitive interactions alongside facilitation. **(3) Stable Community (Cushion loss):** Cushion plants are competitively excluded, leading to their dominance being supplanted by other species, primarily sedges, which form a stable alpine grassland.

Nested within these community stages, we examined finer-level changes at the individual plant level. Here, six degradation substages were defined by the proportional canopy coverage of beneficiary plants relative to the host cushion (Fig. 1): **(1) Within Primary Communities:** Stage 0 comprised bare ground with gravel and semi-weathered substrate; Stage 1 featured healthy cushion plants devoid of beneficiary species (0% cover). **(2) Within Balanced Communities:** Stage 2 and Stage 3 represented cushions with approximately 20% and 50% beneficiary cover, respectively. **(3) Within Stable Communities:** Stage 4 and Stage 5 corresponded to cushions with approximately 80% and 100% beneficiary cover, the latter stage culminating in cushion plant mortality.

Fieldwork was conducted in August 2023 (Zhang et al., 2025c). We established five replicate plots (5 m × 5 m) for each of the three community stages, totaling 15 plots. A composite soil sample (0–10 cm depth), derived from five randomly located sub-samples, was collected from each plot. Concurrently, for the individual-level analysis, we selected five representative cushion plants per substage (total 30 plants). Soil from the center and periphery of each plant was composited into a single sample. All samples were sieved to 2 mm in the field and partitioned for different analyses: one aliquot was air-dried for physicochemical characterization, a second was refrigerated at 4 °C for assaying biological activity, and a third was flash-frozen at −80 °C for subsequent molecular work.

### 2.2 Soil property analyses and microcosm experiment

Prior to the microcosm incubation, a comprehensive set of initial soil properties of each sample was measured. Physicochemical analyses included measurements of pH, total carbon (TC), total nitrogen (TN), total phosphorus (TP), soil organic carbon (SOC), dissolved organic carbon (DOC), ammonium nitrogen (Ammonium), and nitrate nitrogen (Nitrate). Soil microbial biomass for key groups (total bacteria (PLFA_Bacteria), Gram-positive (PLFA_Gp) and Gram-negative bacteria (PLFA_Gn), fungi (PLFA_Fungi), actinomycetes (PLFA_Actinomyces), arbuscular mycorrhizal fungi (PLFA_AMF), and protozoa (PLFA_Protozoa)) was determined by phospholipid fatty acid (PLFA) analysis following standard procedures (Frostegård and Bååth, 1996). The potential activities of extracellular enzymes involved in major nutrient cycles were assessed, including β-glucosidase, sucrase, and cellulase for carbon acquisition; protease for nitrogen acquisition; polyphenol oxidase (PPO) and peroxidase for lignin degradation; and phosphatase for phosphorus mineralization. Soil respiration and nitrogen mineralization rates (NMR) were measured as indicators of general microbial metabolic activity. Detailed protocols for these analyses are described in previous works (Zhang et al., 2024, 2025c, 2025d).

A controlled microcosm experiment was implemented to investigate the effects of key global change scenarios. All soil samples retained their original indigenous bacterial and fungal communities and baseline soil properties collected from the field. The experiment comprised four treatments: **(1) Control (C):** maintained at 15 °C (approximating the optimal daytime temperature during the growing season) and maintained at 50% water-holding capacity (WHC) (Chen et al., 2019). **(2) Warming (T):** maintained at 20 °C (+5 °C above control, simulating the regional warming scenario by the end of this century) and 50% WHC (Stocker et al., 2013). **(3) Dry-Wet Cycling (D):** maintained at 15 °C, with soil moisture fluctuating weekly between 10% WHC (a threshold inducing microbial dormancy) and 50% WHC (Salazar et al., 2018). **(4) Nitrogen Addition (N): maintained at** 15 °C, 50% WHC, with NH_4_NO_3_ added at a rate of 120 kg N ha^−1^ yr^−1^ (reflecting a high-level deposition scenario for the Qinghai-Xizang Plateau) (Hu et al., 2022). The microcosms were established using fresh soil from both community-level and individual-level sampling. This included: (1) community-level: 3 degradation stages × 5 plot replicates = 15 soil samples; (2) individual-level: 3 degradation stages × 2 degradation substages × 5 plant replicates = 30 soil samples. This yielded a total of 45 distinct soil samples. For each of these soil samples, all four treatments were applied. Microcosms consisted of 30 g of fresh soil in 250-ml breathable glass jars. Each unique combination of soil sample and treatment was incubated with three replicate jars (*n*= 3), resulting in a total of 540 microcosm units (45 soil samples × 4 treatments × 3 replicates). After a 60-day incubation period, soil from the three replicate jars pertaining to the same soil sample and treatment was composited into a single sample for DNA extraction and subsequent microbial sequencing. This compositing procedure yielded a final total of 180 samples (45 soil samples × 4 treatments) for microbial community analysis.

### 2.3 Molecular and bioinformatics analyses

Detailed protocols for these analyses are described in our previous work (Zhang et al., 2024, 2025c, 2025d). In brief, total genomic DNA was extracted from soil samples, and the bacterial 16S rRNA gene (V4–V5 region) and fungal ITS2 region were amplified and sequenced on an Illumina NovaSeq 6000 platform. Bioinformatic processing was performed using QIIME2 (Bolyen et al., 2019). Sequences were quality-filtered, denoised, and clustered into amplicon sequence variants (ASVs). Taxonomic classification was assigned using the SILVA 138 database for bacteria and the UNITE 8.3 database for fungi. All samples were rarefied to an even sequencing depth (39,938 sequences per sample for bacteria and 45,280 for fungi) for downstream analysis. Functional guilds of fungal keystone nodes were assigned using FUNGuild (Nguyen et al., 2016), while bacterial keystone functions were predicted using FAPROTAX (Louca et al., 2016).

### 2.4 Microbial network analysis

We constructed microbial co-occurrence networks to characterize potential ecological interactions within bacterial and fungal communities under global change scenarios. It should be noted that, to ensure data comparability, the network structures of the T, N, and D groups were compared based on the control group. All analyses were conducted in R (v.4.4.1) (R Core Team, 2024).

**Network Construction and Validation:** Separate co-occurrence networks were constructed for bacterial and fungal communities across the four experimental treatments (C, D, N and T), resulting in eight distinct networks. We did not construct the cross-domain network because it cannot explore the taxa-dependent nature of the network structure dynamics. To ensure computational efficiency and focus on core taxa, each network was built using the top 500 most abundant amplicon sequence variants (ASVs) per microbial domain, which captured 70.7% and 38.3% of total sequences for fungi and bacteria, respectively (Fig. S1). To validate the robustness of this approach, parallel networks were constructed using the top 1000 ASVs. The resulting topological patterns were generally consistent (Fig. S2–S9), indicating that the network structure was not sensitive to the chosen ASV abundance threshold. The network construction procedure was as follows: pairwise Spearman rank correlation matrices between ASVs were calculated using the cor And P value function from the WGCNA package (Langfelder and Horvath, 2008). Statistically robust associations (edges) were defined by applying stringent filtering criteria: an absolute correlation coefficient |*ρ*| > 0.6 and a Benjamini-Hochberg false discovery rate (FDR)-adjusted *P*< 0.001. The adjacency matrix formed by the filtered correlations was used to build an undirected, weighted network via the igraph package (Csardi and Nepusz, 2006). Finally, all unconnected (isolated) nodes were removed from the network.

**Network Complexity Analysis:** To quantify microbial network complexity, we performed an induced-subnetwork-based analysis for each sample. Specifically, an induced subnetwork was extracted from the corresponding microbial network for each sample based on its ASV composition. Five key metrics were calculated for each of these subnetworks: the number of nodes, the number of edges, average degree, betweenness centrality, and assortativity. Together, these metrics provide a composite quantification of network complexity, encompassing its scale, connection strength, centrality distribution, and link preference. We then analyzed variations in these metrics along two dimensions: (1) by comparing differences across the various global change scenarios, and (2) by examining their trends along the ecosystem degradation stages.

**Network Robustness Assessment:** To evaluate the capacity of microbial networks to withstand node loss, we measured network robustness using two strategies, i.e., random removal and targeted removal scenarios. Herein, network robustness is defined as a network's ability to maintain its overall connectivity when subjected to an external disturbance. This property was quantified by simulating node-removal attacks, which mimic “species loss” (Dunne et al., 2002).

First, for random removal, we simulated the random removal of a progressively increasing proportion of its nodes. At each removal fraction, we conducted 50 random iterations. Following each iteration, two key robustness metrics were recorded: (1) the relative size of the largest connected component (LCC)—the number of nodes in the largest connected subgraph divided by the total number of nodes remaining in the network; and (2) the global efficiency (GE)—a standardized measure of the average inverse shortest path length between all node pairs in the network, reflecting the efficiency of information transfer (Latora and Marchiori, 2001).

Second, for targeted removal, nodes were first classified into four topological roles based on within-module connectivity (Zi) and among-module connectivity (Pi) (Guimerà and Amaral, 2005): peripherals (Zi ≤ 2.5, Pi ≤ 0.62), connectors (Zi ≤ 2.5, Pi > 0.62), module hubs (Zi > 2.5, Pi ≤ 0.62), and network hubs (Zi > 2.5, Pi > 0.62). Nodes were then sequentially removed in descending order of topological importance (|Zi| + Pi), simulating the preferential loss of keystone taxa (Albert et al., 2000). The same robustness metrics (LCC and GE) were recorded at each removal step.

Finally, the area under the curve (AUC) for each robustness index across the entire attack sequence was computed. This AUC value serves as a composite indicator of the overall robustness for each microbial network under random and targeted removal scenarios.

**Analysis of Soil Property Drivers:** To explore the influence of initial soil properties on microbial network complexity, Spearman correlation analysis was conducted between the five sample-level network complexity metrics and a range of initial soil properties. The resulting correlation matrices, after FDR correction, were visualized as heatmaps. This analysis aimed to identify key soil predictors that were most strongly associated with variations in microbial network complexity across different global change scenarios and microbial taxa (bacteria vs. fungi).

**Taxonomic Composition and Keystone Taxa Analysis:** To characterize the taxonomic distinctiveness of microbial communities in this alpine environment, we analyzed phylum-level composition and calculated the proportion of ASVs that could not be classified at the genus level as an indicator of taxonomic novelty. Keystone taxa were defined as module hubs and connectors identified by the Zi-Pi classification described above. To investigate whether keystone taxa serve as biotic drivers of network complexity, the relative abundance of keystone taxa (total and by phylum) was calculated for each sample and correlated with the five network complexity metrics using Spearman's rank correlation. All correlations were FDR-corrected for multiple comparisons.

## 3 Results

### 3.1 Taxon-specific shifts in microbial network robustness and complexity under global change scenarios

Microbial co-occurrence network analyses revealed that soil fungal and bacterial communities responded to the global change scenarios (C, D, N and T) in taxon-specific ways (Fig. 2). Under random node removal, the two microbial groups exhibited distinct robustness patterns characterized by different baseline levels and robustness dynamics. For fungal networks, the robustness (i.e., GE and LCC) AUC values under the D, N, and T scenarios were higher than under the C scenario (Fig. 2C and D). The robustness curves showed a gradual, near-linear decline throughout the removal sequence, with relatively low baseline robustness (LCC-AUC: 0.355**–**0.434, GE-AUC: 0.062**–**0.085). In contrast, bacterial networks maintained substantially higher baseline robustness (LCC-AUC: 0.916**–**0.935, GE-AUC: 0.690**–**0.723) and showed scenario-dependent responses: the D scenario yielded the highest robustness, whereas N and T scenarios showed no increase compared to C (Fig. 2E and F). Notably, bacterial robustness curves exhibited threshold-like dynamics—remaining near initial connectivity until approximately 80% of nodes were removed, after which they collapsed sharply. These scenario-dependent patterns were broadly consistent under targeted removal (Table S3 and Fig. S10).

**Fig. 2:**
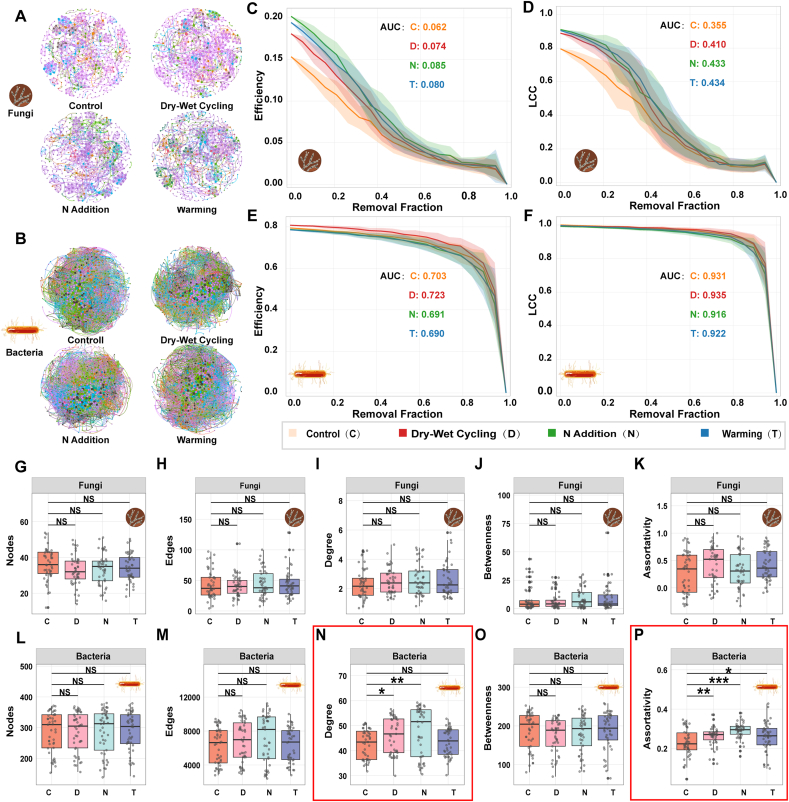
**Effects of global change scenarios on microbial network complexity and robustness.** (A–B) Co-occurrence networks of fungal (A) and bacterial (B) communities under four scenarios: Control (C), Dry-wet cycling (D), Nitrogen addition (N), Warming (T), and (C–F) Network robustness under simulated species loss: Global Efficiency (GE) and the relative size of the Largest Connected Component (LCC) of fungal (C–D) and bacterial (E–F) networks. (G–P) Network complexity metrics across scenarios: the number of nodes, the number of edges, average degree, betweenness centrality, and assortativity for fungal (G–K) and bacterial (L–P) networks. In C–F, the lines and shaded areas represent the mean trend and standard deviation across replicates, respectively. The area under the curve (AUC) quantifies overall robustness. Asterisks indicate significant differences from the control scenarios (NS, not significant; ∗*P*< 0.05; ∗∗*P*< 0.01; ∗∗∗*P*< 0.001). Red boxes highlight key metrics of particular interest.

To further characterize the mechanisms underlying these taxon-specific robustness patterns, we performed targeted removal simulations that sequentially eliminated nodes based on their topological importance (Table S3, Fig. S10). This analysis revealed that fungal and bacterial networks employ fundamentally different strategies to maintain stability. Fungal network robustness declined substantially under targeted compared to random removal (LCC-AUC decreasing by 34–52%), indicating a hub-dependent structure where stability relies on a few highly connected nodes. In contrast, bacterial network robustness showed negligible sensitivity to removal strategy (< 3% change in LCC-AUC), reflecting connector-mediated redundancy that distributes connectivity across the network. Consistent with these topological differences, fungal networks contained 1–9 module hubs per treatment, whereas bacterial networks lacked hubs entirely but possessed 30–63 connectors (Fig. S11). These contrasting topological strategies align with their distinct taxonomic compositions: fungal communities were dominated by stress-tolerant Ascomycota (68.8%; Fig. S12), while bacterial communities exhibited higher taxonomic novelty, with 70.2% of ASVs unresolved at the genus level (Fig. S13).

In addition to robustness, we examined network complexity responses to global change scenarios. Fungal network complexity (i.e., the number of nodes, edges, average degree, betweenness centrality, and assortativity) showed no significant change across scenarios relative to C (Fig. 2G–K), indicating structural stability under environmental stress. Bacterial network complexity, however, was altered under specific scenarios: average degree increased under D and N (Fig. 2N), and assortativity increased under D, N, and T, with the greatest increase observed under N (Fig. 2P).

### 3.2 Asymmetric responses of microbial networks to the degradation stages

We further analyzed the dynamics of microbial co-occurrence networks along the sequential cushion plant degradation stages. We found that the complexity of both fungal and bacterial networks showed stage-dependent asymmetric responses. At the individual-level degradation, fungal network complexity increased rapidly from Stage 0 to Stage 1, with significant and synchronous rises in the number of nodes, the number of edges, and betweenness centrality. No significant changes in complexity metrics were observed from Stage 1 to Stage 3. A second change occurred at Stage 3–4, where the number of edges and average degree increased significantly. At the community-level degradation, network complexity decreased from the primary to the balanced community stage, showing significant reductions in the number of edges and average degree. However, a distinct change was observed during the transition to the stable community. Here, the number of nodes decreased significantly, whereas the number of edges, average degree, and betweenness centrality increased synchronously, forming a more interconnected and centralized network (Fig. 3A–E).

**Fig. 3:**
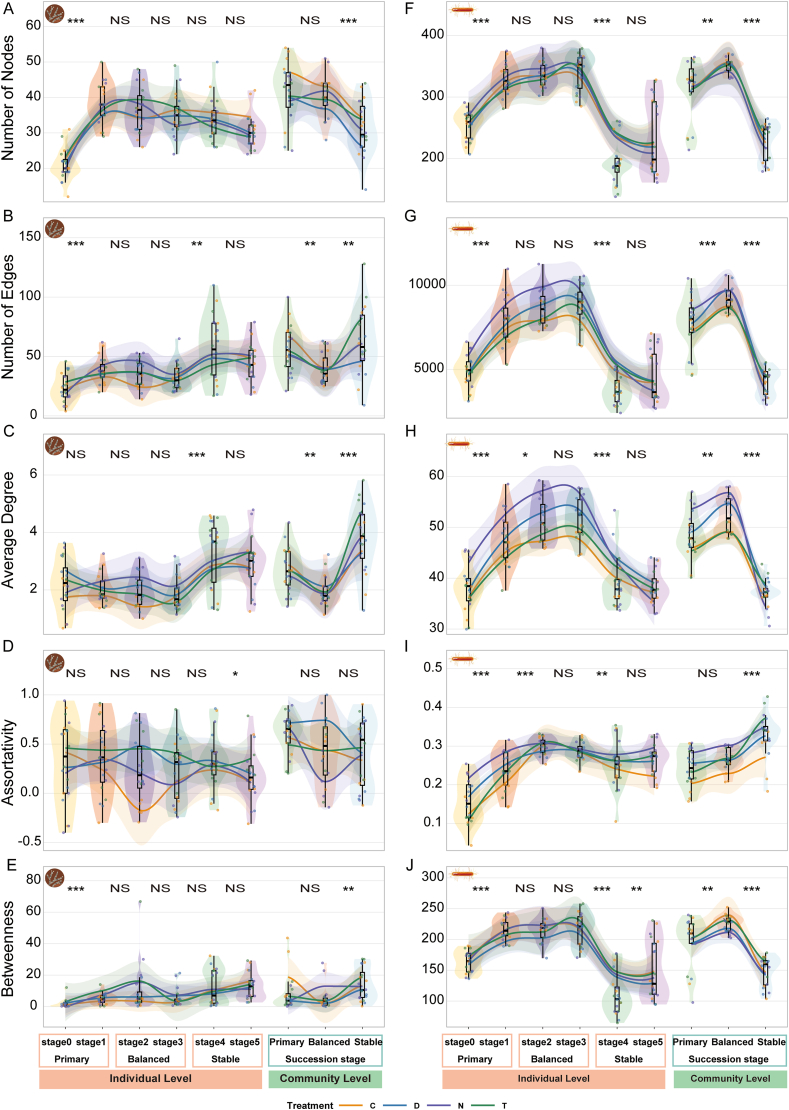
**Dynamics of network complexity metrics in fungal and bacterial co-occurrence networks along degradation stages.** Panels A–E show fungal networks, and panels F–J show bacterial networks. Five key complexity metrics are presented: the number of nodes (A, F), the number of edges (B, G), average degree (C, H), assortativity (D, I), and betweenness centrality (E, J). The x-axis represents the degradation gradient, integrating six individual degradation stages (Stage 0–Stage 5) nested within three broader community degradation stages (Primary, Balanced, Stable). The colored dots represent samples from different global change scenarios: Control (C, orange), Dry-wet cycling (D, blue), Nitrogen addition (N, purple), and Warming (T, green). The solid colored lines indicate LOESS fit curves for each treatment (span = 0.8), and the shaded ribbons indicate the corresponding 95% confidence intervals. LOESS fitting was performed separately for the individual-level (Stage 0–Stage 5) and community-level (Primary, Balanced, Stable) sequences. Asterisks mark significant differences between adjacent stages, as determined by the Wilcoxon rank-sum test (NS, not significant; ∗*P* < 0.05, ∗∗*P* < 0.01, ∗∗∗*P* < 0.001).

In contrast, bacterial networks displayed divergent trajectories at these key stages (Fig. 3F–J). At the individual-level degradation, all complexity metrics (the number of nodes, the number of edges, average degree, betweenness centrality, and assortativity) increased significantly from Stage 0–1. However, from Stage 3–4, all these metrics declined significantly. At the community-level degradation, the transition from the primary to the balanced community resulted in significant increases in four complexity metrics (the number of nodes, the number of edges, average degree, and betweenness centrality), while assortativity did not change significantly. Conversely, the subsequent shift from the balanced to the stable community was marked by a significant decrease in network complexity, with reductions in the same four metrics accompanied by an increase in assortativity. Consequently, at two critical stages—from Stage 3 to Stage 4 at the individual-level, and from the balanced to the stable community at the community-level—fungal and bacterial networks exhibited divergent trajectories: reorganization vs simplification. This asymmetric response pattern underscores a fundamental difference in how these two microbial taxa respond to ecosystem degradation and implies the existence of degradation stage-specific thresholds.

### 3.3 Abiotic and biotic predictors of network dynamics

**Abiotic predictors.** Spearman correlation analysis revealed that the global change scenarios (C, T, D and N) profoundly modulated the associations between soil microbial network complexity and initial soil properties. Fungal and bacterial networks responded in an opposing manner (Fig. 4). Under the C scenario, fungal complexity metrics showed only limited significant correlations with some soil properties. However, the T and N scenarios markedly strengthened these associations, resulting in 73 significant positive correlations (Fig. 4A). Specifically, fungal complexity metrics, such as average degree, betweenness centrality, and the number of edges, were positively correlated with soil nutrients (e.g., TP, TN, TC, SOC), microbial biomass (e.g., PLFA_Bacteria, PLFA_Fungi, PLFA_AMF), extracellular enzyme activities (e.g., β-glucosidase), and metabolic activity indicators (e.g., NMR). A particularly strong positive correlation was observed under the N scenario between fungal betweenness centrality and SOC (*ρ*= 0.707, *P* < 0.001). In contrast, the D scenario weakened these associations, yielding mostly non-significant correlations.

**Fig. 4:**
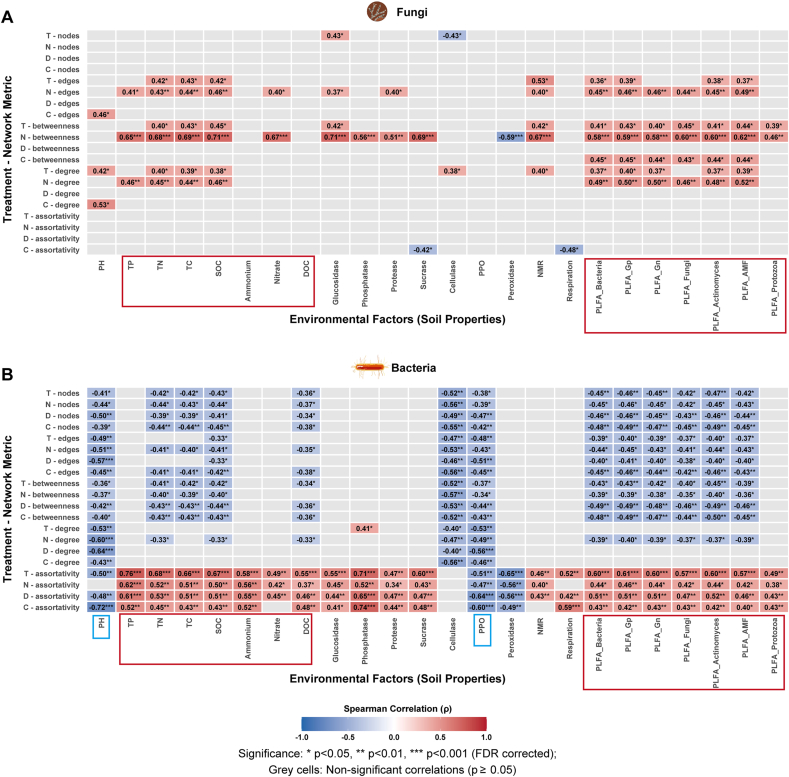
**Spearman rank correlations between initial soil properties and microbial co-occurrence network complexity under microcosm treatments.** (A) Fungal networks. (B) Bacterial networks. Each cell displays the correlation coefficient (*ρ*) between a network complexity metric (y-axis) and an initial soil property (x-axis) for each treatment: Control (C), Warming (T), Nitrogen addition (N), and Dry-wet cycling (D). The color scale denotes the strength and direction of correlation, with red indicating positive and blue indicating negative correlations. Only statistically significant correlations (FDR-corrected *P* < 0.05) are colored. Non-significant correlations (*P* ≥ 0.05) are shown in grey. Significance levels are denoted as follows: ∗*P* < 0.05, ∗∗*P* < 0.01, ∗∗∗*P* < 0.001. Abbreviations: PH (Soil pH), TP (Total Phosphorus), TN (Total Nitrogen), TC (Total Carbon), SOC (Soil Organic Carbon), Ammonium (NH_4_^+^-N), Nitrate (NO_3_^-^-N), DOC (Dissolved Organic Carbon), Glucosidase (β-glucosidase activity), Phosphatase (Phosphatase activity), Protease (Protease activity), Sucrase (Sucrase activity), Cellulase (Cellulase activity), PPO (Polyphenol oxidase activity), Peroxidase (Peroxidase activity), NMR (Nitrogen mineralization rate), Respiration (Soil basal respiration), PLFA_Bacteria (Total bacterial biomass), PLFA_Gp (Gram-positive bacterial biomass), PLFA_Gn (Gram-negative bacterial biomass), PLFA_Fungi (Total fungal biomass), PLFA_Actinomyces (Actinomycetes biomass), PLFA_AMF (Arbuscular mycorrhizal fungi biomass), PLFA_Protozoa (Protozoan biomass). Red boxes highlight key soil properties of particular interest.

Bacterial networks exhibited pervasive negative correlations with initial soil properties, while assortativity showed a positive response. Most of their complexity metrics (the number of nodes, the number of edges, average degree, betweenness centrality) showed widespread and significant negative correlations with soil pH, soil nutrients (e.g., TP, TN, TC, SOC), microbial biomass (e.g., PLFA_Bacteria, PLFA_Fungi, PLFA_AMF), and extracellular enzyme activities (e.g., cellulase) across all scenarios (Fig. 4B). For instance, bacterial average degree correlated strongly negatively with pH under D scenario (*ρ* = *-*0.635, *P* < 0.001). Notably, the N scenario specifically intensified this negative correlation pattern, yielding the highest number of significant negative correlations (*n* = 52), whereas the numbers of significant negative correlations under the T and D scenarios were similar to that under the C scenario.

Taken together, both soil microbial biomass and soil nutrients served as key common predictors of network responses. The biomass of various microbial groups quantified by PLFA analysis (e.g., PLFA_Bacteria, PLFA_Gp, PLFA_Fungi) showed strong predictive power for both fungal and bacterial networks, with their significant correlations accounted for 48.2% and 40.9% of all significant correlations within each respective network. Soil nutrients (e.g., TN, TC, SOC) also demonstrated considerable predictive ability, contributing 30.6% and 27.4% of the significant correlations in fungal and bacterial networks, respectively. Furthermore, soil pH and PPO activity were also important predictors for bacterial networks, showing significant correlations with nearly all complexity metrics across all four global change scenarios.

**Biotic predictors.** Beyond these abiotic constraints, the abundance of keystone taxa (module hubs and connectors) emerged as a significant biotic predictor of network dynamics (Fig. S14–S15, Table S4). For bacteria, total keystone taxa abundance showed strong positive correlations with network complexity: Nodes (*ρ* = 0.63, *P*< 0.001), Edges (*ρ* = 0.71, *P*< 0.001), average degree (*ρ* = 0.70, *P*< 0.001), and betweenness centrality (*ρ* = 0.54, *P* < 0.001). Among bacterial phyla, Actinobacteriota (*ρ* = 0.72–0.79) and Proteobacteria (*ρ* = 0.70–0.79) exhibited the strongest positive associations with complexity metrics, while Gemmatimonadota showed the highest correlation with betweenness centrality (*ρ* = 0.75, *P* < 0.001). Notably, Methylomirabilota was the only phylum showing consistent negative correlations with all complexity metrics (*ρ* = −0.65 to −0.71, *P* < 0.001). Interestingly, Acidobacteriota—despite harboring numerous keystone nodes (*n* = 18, ranking third among phyla)—showed no significant correlation with network complexity, suggesting a role as structural scaffolding rather than active drivers (Fig. 5, Fig. S16).

**Fig. 5:**
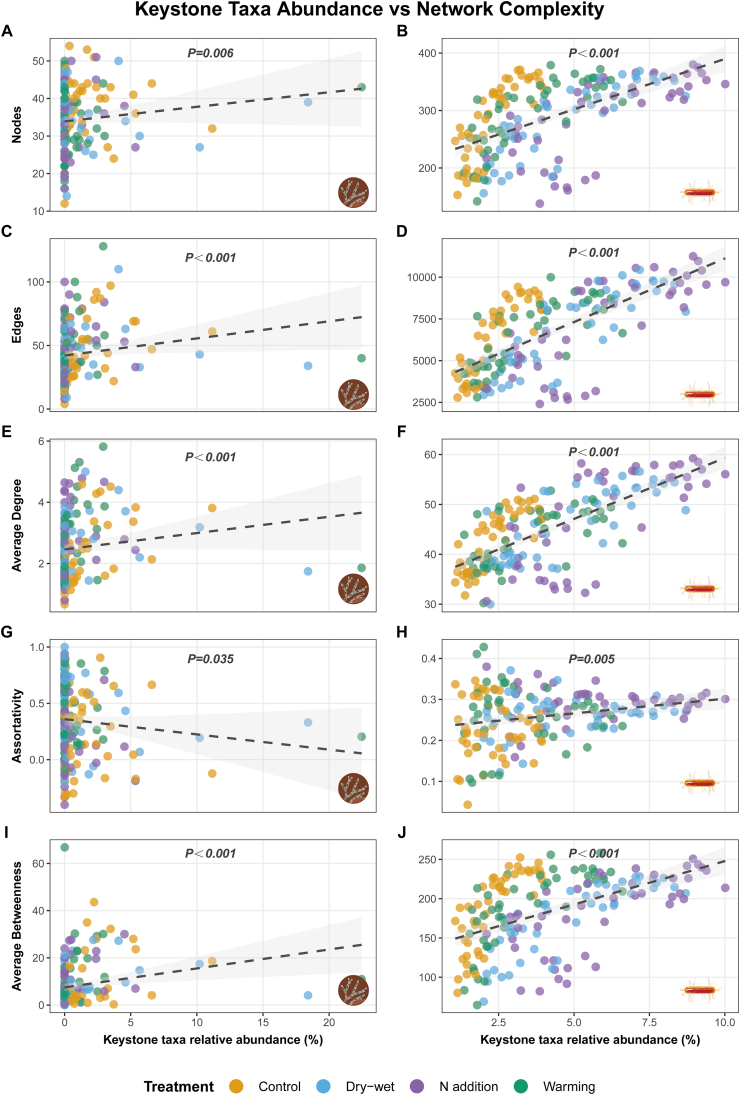
**Scatter plots of keystone taxa abundance versus network complexity metrics.** Scatter plots showing relationships between keystone taxa relative abundance and network complexity metrics for fungi and bacteria. Each point represents one sample, colored by treatment: Control (orange), Dry-wet cycling (light blue), Nitrogen addition (purple), and Warming (green). Dashed lines indicate linear regression fits with 95% confidence intervals (shaded areas). Significance levels are shown for each metric. For bacteria, keystone abundance showed strong positive correlations with Nodes (*ρ* = 0.63, *P* < 0.001), Edges (*ρ* = 0.71, *P* < 0.001), Average Degree (*ρ* = 0.70, *P* < 0.001), and Average Betweenness (*ρ* = 0.54, *P* < 0.001), but weaker correlation with Assortativity (*ρ* = 0.21, *P* < 0.01). For fungi, correlations were moderate for Edges (*ρ* = 0.40) and Average Betweenness (*ρ* = 0.46), with a weak negative correlation for Assortativity (*ρ* = −0.16). *n* = 180 samples per panel.

For fungi, keystone taxa abundance showed moderate positive correlations with Edges (*ρ* = 0.40, *P* < 0.001) and betweenness centrality (*ρ* = 0.46, *P* < 0.001). Ascomycota keystone abundance was specifically associated with network edges (*ρ* = 0.37, *P* < 0.001) and betweenness (*ρ* = 0.38, *P* < 0.001). Treatment-specific analysis revealed that bacterial keystone-network correlations remained strong across most scenarios but were attenuated under warming (Fig. 5, Fig. S16), suggesting that thermal stress decouples the biotic regulation of network structure.

## 4 Discussion

### 4.1 Context-dependent robustness strategies: contrasting structures in fungal and bacterial networks

Our results reveal that fungal and bacterial networks adopt fundamentally different strategies to maintain stability, and their relative robustness depends on the type of removal strategy. Under random node removal, bacterial networks showed substantially higher baseline robustness than fungal networks (LCC-AUC: 0.916–0.935 vs. 0.355–0.434). Bacterial networks maintained near-complete connectivity until roughly 80% of nodes were removed, then collapsed abruptly; fungal networks, by contrast, declined steadily throughout the removal sequence (Fig. 2C–F). Yet when we examined responses to global change scenarios, the pattern reversed: fungal robustness increased under D, N, and T relative to C, whereas bacterial robustness rose only under D and remained unchanged under N and T.

Targeted removal simulations uncovered the structural basis for these contrasting patterns. When topologically important nodes were removed preferentially, fungal network robustness dropped sharply (LCC-AUC declining by 34–52% compared to random removal), revealing a hub-dependent structure in which stability hinges on a few highly connected module hubs (1–9 per treatment). Bacterial networks, however, showed negligible sensitivity to removal strategy (< 3% change in LCC-AUC). They lacked hubs entirely but contained 30–63 connectors per treatment, distributing inter-module links across many nodes rather than concentrating them in vulnerable keystones (Fig. S10). This distributed design explains why bacterial networks tolerate random loss so well, yet struggle to reconfigure adaptively: without dominant hubs to reorganize around, they lack the structural flexibility to strengthen cohesion under novel conditions **(**Albert et al., 2000**)**.

These topological strategies are rooted in distinct taxonomic profiles. Fungal communities were overwhelmingly dominated by Ascomycota (68.8%), a phylum known for stress-tolerant traits such as thick-walled spores, melanized hyphae, and versatile extracellular enzymes (Egidi et al., 2019). The positive correlations we observed between fungal network complexity and β-glucosidase activity (Fig. 4A) support this link: taxa capable of flexible carbon acquisition may occupy hub positions and thereby stabilize the network. Bacterial communities, in contrast, exhibited profound taxonomic novelty, with 70.2% of ASVs unresolved at the genus level—well above typical values for temperate soils (Delgado-Baquerizo et al., 2018). This microbial “dark matter” (Bernard et al., 2018) suggests that the connector-rich topology emerges from diverse, undescribed lineages that maintain inter-module bridges through metabolic complementarity rather than competitive dominance.

The scenario-specific responses we observed are also consistent with these structures. Bacterial assortativity rose markedly under N, yet robustness did not follow. This decoupling likely reflects environmental filtering: nitrogen addition favors taxa with similar tolerances, homogenizing connections and raising assortativity, but simultaneously reducing the diversity of ecological strategies that underpin robustness (Leff et al., 2015). Fungal networks, by comparison, maintained stable complexity while enhancing robustness, suggesting that their hub-centric design allows adaptive strengthening without wholesale structural reorganization. Negative bacterial responses to nitrogen enrichment have been reported elsewhere on the Qinghai-Xizang Plateau (Liu et al., 2025), and our findings clarify the network-level mechanism involved.

The patterns documented here both parallel and extend observations from other high-elevation or cold systems. In the European Alps, soil microbial networks along elevation gradients likewise display hub-dominated fungal modules and connector-rich bacterial structures (Karimi et al., 2018). Permafrost soils in Arctic tundra show analogous threshold-like bacterial collapse under simulated thaw (Mackelprang et al., 2011). However, the degree of taxonomic novelty in our bacterial communities (> 70% unresolved at genus level) exceeds values reported for most alpine and Arctic sites, underscoring the distinctive nature of the Qinghai-Xizang microbiome and the need for region-specific functional characterization. At the same time, the convergent network structure patterns across continents suggest that hub-dependence in fungi and connector-mediated redundancy in bacteria may represent broadly conserved organizational strategies in cold, resource-limited soils.

### 4.2 Threshold responses along degradation stages: early-warning signals from microbial networks

Our analysis of network dynamics along the cushion plant degradation sequence revealed pronounced nonlinearity. Rather than declining gradually, network complexity shifted abruptly at specific stages, consistent with the theory of critical transitions in ecosystems (Scheffer et al., 2009). At two key junctures—Stage 3 to 4 at the individual level, and the balanced-to-stable community transition—fungal and bacterial networks diverged sharply (Fig. 3). Fungal networks moved toward a more interconnected state: node numbers fell while edge counts and betweenness centrality rose, indicating network reorganization. Bacterial networks, by contrast, showed synchronous declines across multiple complexity metrics, pointing to network simplification. This asymmetry suggests that cushion plant degradation creates ecological conditions that facilitate fungal network rewiring while disrupting bacterial network integrity.

Why do fungal networks reorganize while bacterial networks simplify? The hub-dependent structure of fungal networks may confer reorganizational capacity: as peripheral nodes are lost, surviving hubs can recruit new connections and maintain cohesion. Bacterial networks, lacking such organizational centers, appear to lose connectivity in a more diffuse, irreversible fashion. This interpretation aligns with threshold dynamics reported in other sensitive ecosystems, including Arctic permafrost (Turetsky et al., 2020) and dryland soils (Delgado-Baquerizo et al., 2016b). Our data thus support the view that microbial network complexity can serve as an early indicator of ecosystem state change.

Critically, these network-based signals may precede shifts detectable by conventional metrics. In our degradation gradient, microbial network complexity exhibited clear threshold responses at Stage 3–4 and during the balanced-to-stable transition, whereas soil respiration and enzyme activities changed more gradually (Zhang et al., 2025c). Network topology captures emergent properties of community organization—interaction rewiring, keystone redistribution—that manifest before aggregate functional outputs decline. This temporal precedence implies that network monitoring could provide a valuable “lead time” for management, flagging community destabilization while ecosystem functions remain within acceptable bounds (Scheffer et al., 2012). Compared with traditional indicators such as vegetation cover, microbial network metrics offer a window into the hidden dynamics of belowground communities, though they require molecular tools and analytical expertise that may limit routine application.

### 4.3 Abiotic and biotic predictors of network dynamics

Our correlation analyses confirm that initial soil properties shape microbial network complexity in a taxa-specific manner (Wardle et al., 2004). Under N and T scenarios, fungal network metrics correlated positively with soil nutrients and microbial biomass (Fig. 4A), reflecting a “resource-responsive” mode: where resources are abundant, fungi form denser, more centralized networks. The strong link between fungal betweenness centrality and SOC under N (*ρ* = 0.707) underscores the role of carbon availability in supporting hub formation. Bacterial networks, however, displayed pervasive negative correlations with the same properties across all scenarios. A compensatory pattern emerged for assortativity, which remained positively correlated with soil nutrients. This divergence hints at a stress-driven adjustment: under harsher conditions (lower nutrients, lower biomass), bacterial communities may shift toward more assortative structures—strengthening ties among ecologically similar taxa to preserve core sub-networks even as overall complexity declines (Coyte et al., 2015).

Initial microbial biomass (PLFA_Bacteria, PLFA_Fungi, PLFA_AMF) and soil nutrients (TN, TC, SOC) emerged as the most consistent abiotic predictors, together accounting for roughly half of all significant correlations in both fungal and bacterial networks. Biomass likely represents the energetic base available for network assembly; nutrients provide the raw materials. For bacteria specifically, pH and PPO activity served as additional predictors: low pH inhibits bacterial enzyme function (Rousk et al., 2010), while elevated PPO signals a shift toward recalcitrant carbon processing (Sinsabaugh, 2010). Both factors act as environmental filters that constrain the assembly of complex bacterial networks.

Beyond abiotic controls, keystone taxa themselves emerged as biotic drivers of network structure—a dimension often overlooked when only abiotic environmental variables are considered (Fig. S14–S15, Table S4). For bacteria, the total abundance of keystone taxa (module hubs and connectors) correlated strongly with complexity metrics (*ρ* = 0.63–0.71), rivaling or exceeding the predictive power of any single soil property. Among phyla, Actinobacteriota and Proteobacteria—groups known for metabolic versatility—showed the strongest positive associations with complexity, consistent with their role as generalists that sustain diverse inter-taxa links. Gemmatimonadota, though less abundant, displayed the highest correlation with betweenness centrality, suggesting a disproportionate role in network cohesion. Interestingly, Acidobacteriota harbored numerous keystone nodes (*n* = 18, the third highest among phyla) yet showed no correlation with overall complexity. This paradox implies that Acidobacteriota keystones function as “structural scaffolding”—maintaining structure through persistence rather than proliferation—consistent with their oligotrophic, slow-growing lifestyle (Kielak et al., 2016). For fungi, keystone abundance correlated more modestly with network metrics, and Ascomycota keystones were specifically linked to edge number and betweenness.

Environmental context modulated the strength of biotic regulation. Bacterial keystone—complexity correlations remained robust under most scenarios but weakened substantially under warming (Fig. S16). This thermal decoupling suggests that elevated temperature disrupts the link between keystone demography and network organization, potentially by accelerating community turnover or altering interaction strengths in ways that override the stabilizing influence of keystone taxa **(**Zhou et al., 2016**)**. Functionally, the observation that roughly 90% of bacterial keystone nodes lacked database annotations highlights the vast “dark matter” within this alpine microbiome. These uncharacterized keystones may harbor metabolic capabilities critical for ecosystem function, underscoring the need for culture-independent functional profiling of high-elevation soils.

### 4.4 Implications for ecosystem management: from network mechanisms to actionable strategies

The taxa-specific responses documented here offer a microbe-centric lens for managing alpine tundra degradation, complementing vegetation-based approaches.

Prioritizing bacterial network integrity: bacterial networks did not gain robustness under nitrogen addition or warming, and their complexity declined at key degradation stages. Because bacteria mediate rapid nutrient turnover, this vulnerability threatens fundamental soil functions (Delgado-Baquerizo et al., 2016a; Falkowski et al., 2008). Management should therefore focus on limiting nitrogen inputs and mitigating thermal stress in sensitive areas. Fungal networks, with their adaptive hub-dependent design, provide a stabilizing asset, but they cannot substitute for bacterial–driven processes.

Defining intervention thresholds: our data identify quantitative benchmarks for early action. At the individual plant scale, the transition from Stage 3 (roughly 50% beneficiary cover on cushion canopies) to Stage 4 (80% cover) marks the point at which bacterial complexity drops and fungal networks begin reorganizing. In practical terms, restorative measures—removing encroaching beneficiaries, excluding grazers, or reducing trampling—should be initiated before beneficiary cover exceeds 50% on individual cushions. At the community scale, plots where cushion plants account for less than about 30% of total vegetation cover may be flagged for intensified monitoring and intervention.

Establishing predictive soil indicators: the strong correlations between initial soil conditions and subsequent network dynamics enable risk-based prioritization. Based on our threshold analysis, sites where soil pH declines from healthy baselines (∼6.0) toward the 5.5–5.7 range should be prioritized for monitoring, as this range corresponds to the critical transition zone identified in our network dynamics. PPO activity showed scale-dependent patterns during these transitions and should be interpreted in conjunction with other indicators rather than as a standalone threshold. Incorporating these thresholds into routine soil surveys would allow managers to identify vulnerable microsites before visible plant degradation occurs.

Leveraging keystone taxa: keystone abundance directly predicts network complexity, implying that conserving these topologically important taxa is as important as managing abiotic conditions. Although most bacterial keystones remain uncharacterized, their ecological roles—likely involving metabolic bridging between modules—can be supported indirectly by maintaining habitat heterogeneity and avoiding homogenizing disturbances such as heavy nitrogen fertilization.

Scope of applicability: the framework developed here applies most directly to cushion-plant-dominated alpine tundra undergoing competitive displacement—a pattern widespread across the Qinghai-Xizang Plateau and analogous alpine systems worldwide (Cavieres et al., 2013). The specific numerical thresholds (e.g., 50% beneficiary cover, pH < 5.5) are empirically derived from our study site and should be validated locally before extrapolation. The underlying principles—taxa-specific network vulnerability, stage-based intervention windows, and soil-property-based risk assessment—are more likely to generalize, but their quantitative application will require site-specific calibration.

### 4.5 Methodological considerations and study limitations

The microcosm approach employed here, while enabling controlled treatment comparisons, inherently simplifies real-world ecological interactions. Microcosms exclude plant–soil feedbacks, faunal interactions, and the spatial heterogeneity of natural ecosystems (Fraser et al., 2012). The 60-day incubation, though sufficient to detect network restructuring, may not capture longer-term succession or seasonal dynamics. Our findings should therefore be interpreted as revealing the intrinsic response potential of these microbial communities. Field experiments incorporating in situ warming, nitrogen addition, and precipitation manipulation are needed to confirm that the patterns observed here hold under realistic environmental complexity.

Co-occurrence network analysis, while effective for revealing community-wide association patterns, cannot distinguish direct ecological interactions from indirect associations driven by shared environmental responses (Faust and Raes, 2012). The structures we report thus represent statistical associations, not verified interaction networks. Additionally, compositional data (relative abundances) can introduce spurious correlations (Gloor et al., 2017). Although we applied stringent thresholds (|*ρ*| > 0.6, FDR < 0.001), some edges may still be methodological artifacts. Time-series sampling, experimental manipulation of candidate keystones, or metabolic modeling could strengthen causal inference in future work.

The high taxonomic novelty of bacterial communities (70.2% of ASVs unclassified at the genus level), while highlighting the distinctiveness of alpine soil microbiomes, constrains the functional interpretation of our results. While this “dark matter” highlights the novelty of alpine soil microbiomes, it also means that many topologically important taxa cannot be linked to known traits. Metagenomic or metatranscriptomic approaches would enable functional characterization of these uncharacterized keystones and clarify the metabolic underpinnings of their network roles.

Caution is needed when generalizing these results, as they are derived from a single alpine tundra site with one specific cushion plant degradation trajectory. The mechanistic principles we describe—hub-dependent fungal network structures, connector-mediated bacterial redundancy, stage-specific thresholds—may apply broadly to stress-gradient ecosystems, but the specific quantitative patterns are context-dependent. Replication across multiple sites, vegetation types, and climatic gradients is necessary before these results can be translated into universal management prescriptions for alpine systems.

## 5 Conclusion

By integrating field surveys across degradation stages with controlled microcosm experiments, this study reveals taxa-specific and nonlinear responses of soil microbial networks to global change scenarios and cushion plant degradation in alpine tundra. The key findings are:

(1) Microbial network responses are taxa-specific and structurally determined. Fungal networks displayed resilience through a hub-dependent structure: they maintained complexity without significant change and enhanced robustness under various global change scenarios. However, targeted removal simulations revealed their vulnerability to keystone loss, with robustness declining sharply (34–52%) when topologically important nodes were preferentially removed. Bacterial networks exhibited connector-mediated redundancy, conferring high baseline robustness but limited adaptive capacity; their robustness did not increase under warming or nitrogen addition, while assortativity increased under nitrogen addition.
(2) Across degradation stages, network complexity showed nonlinear and asymmetric shifts, indicating critical thresholds. At key stages, fungal networks reorganized via node reduction and connection strengthening, whereas bacterial networks simplified. This asymmetric response provides an early-warning signal for ecosystem degradation. Notably, these network-based signals may precede changes detectable by conventional ecosystem indicators, offering a critical lead time for management intervention.
(3) Both abiotic and biotic factors mediated network complexity in a taxa- and scenario-dependent manner. Among abiotic predictors, fungal network complexity correlated positively with soil nutrients and microbial biomass, especially under nitrogen addition and warming. Conversely, most bacterial complexity metrics were negatively correlated with these properties, while assortativity showed a positive response. Initial microbial biomass, soil pH, and PPO activity were key predictors of bacterial network dynamics. Beyond abiotic factors, keystone taxa abundance emerged as a significant biotic driver of bacterial network complexity, with correlations rivaling those of soil properties. However, this biotic regulation was attenuated under warming, suggesting that thermal stress decouples keystone–network relationships.

These insights support a vulnerability-based management framework for alpine tundra. This framework prioritizes protecting bacterial network integrity by mitigating warming and nitrogen deposition, utilizes the identified pre-threshold stage as a critical intervention window, and integrates soil and microbial indicators for early warning and targeted restoration.

## Authors' contributions

Y.Z.Z.: Conceptualization, Investigation, Methodology, Writing-review & editing, Supervision. W.G.S.: Conceptualization, Funding acquisition, Resources, Supervision, Writing-review & editing. M.D.Y.: Investigation, Formal analysis, Visualization, Writing-original draft. Y.J.Y.: Investigation, Data curation. X.L.: Investigation, Data curation. W.Y.X.: Investigation, Data curation. S.J.L.: Investigation, Data curation. H.S.: Funding acquisition, Resources, Supervision. All authors have read and agreed to the published version of the manuscript.

## Data availability

The raw datasets generated during the current study are available in the Science Data Bank (https://cstr.cn/31253.11.sciencedb.16922). All methods and processes can be obtained directly from the online databases or publications described in the Methods.

## Declaration of competing interest

The author Yazhou Zhang is an Editorial Board Member for Plant Diversity and was not involved in the editorial review or the decision to publish this article. The other authors declare that they have no known competing financial interests or personal relationships that could have appeared to influence the work reported in this paper.

## Figures and Tables

**figs1:**
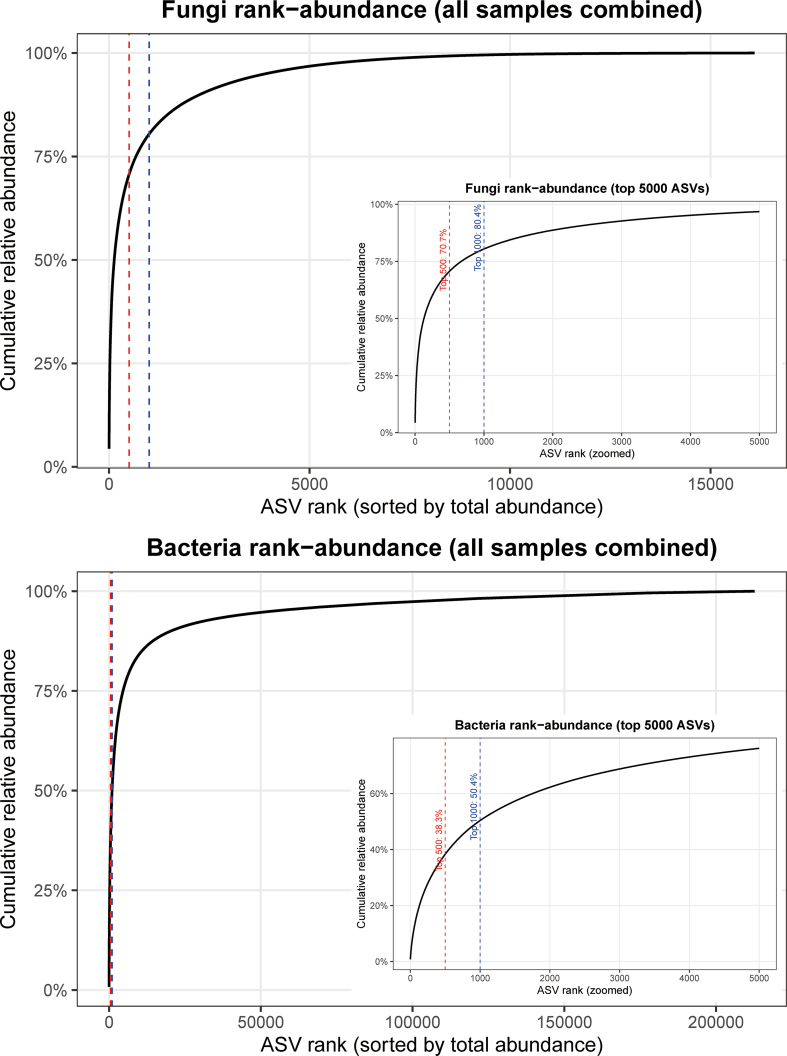


**figs2:**
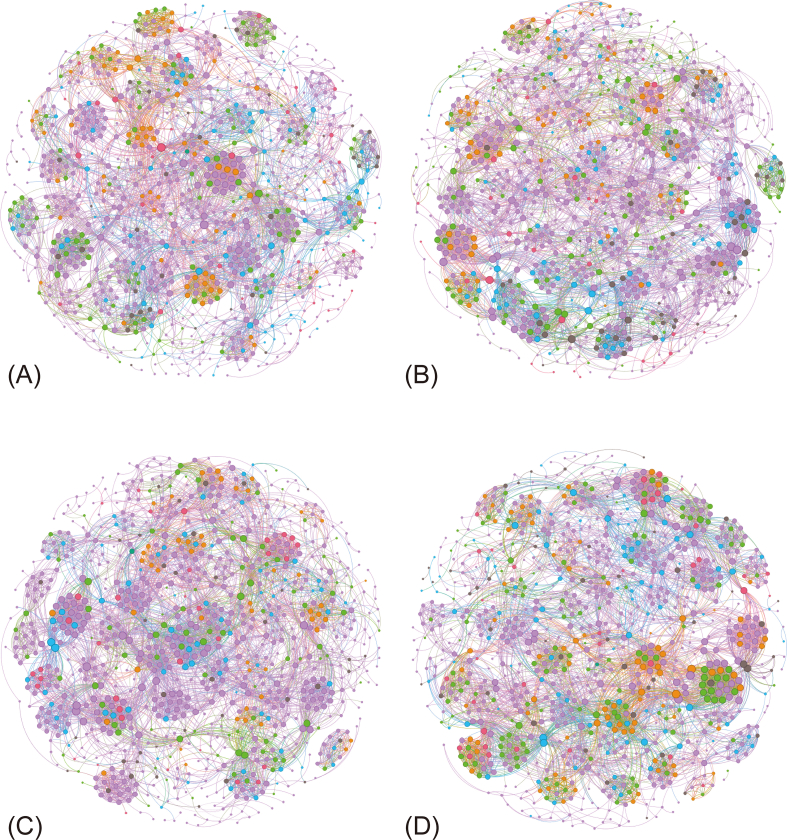


**figs3:**
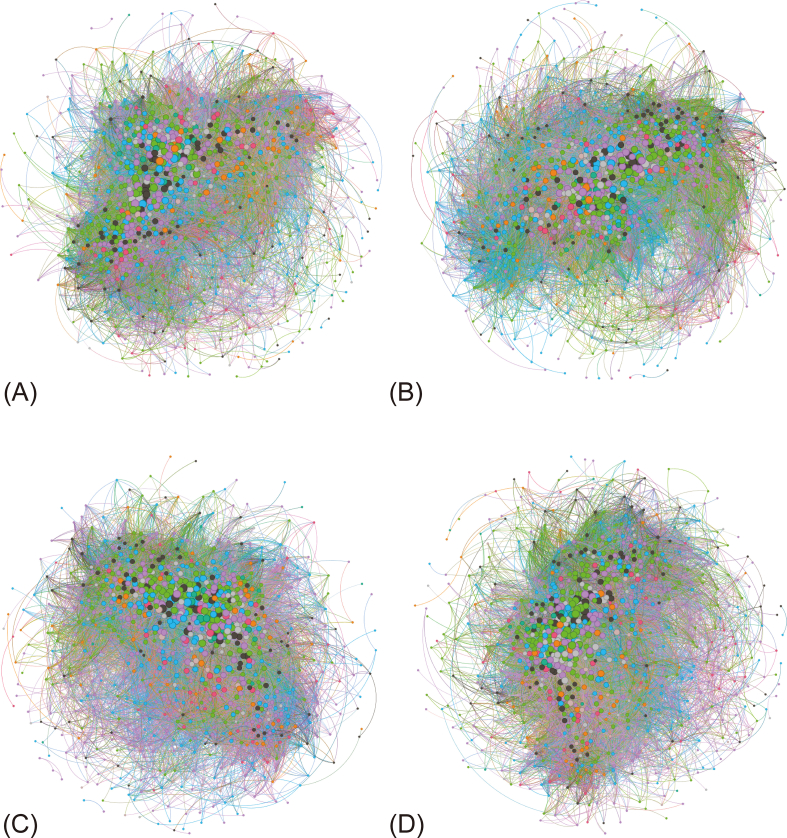


**figs4:**
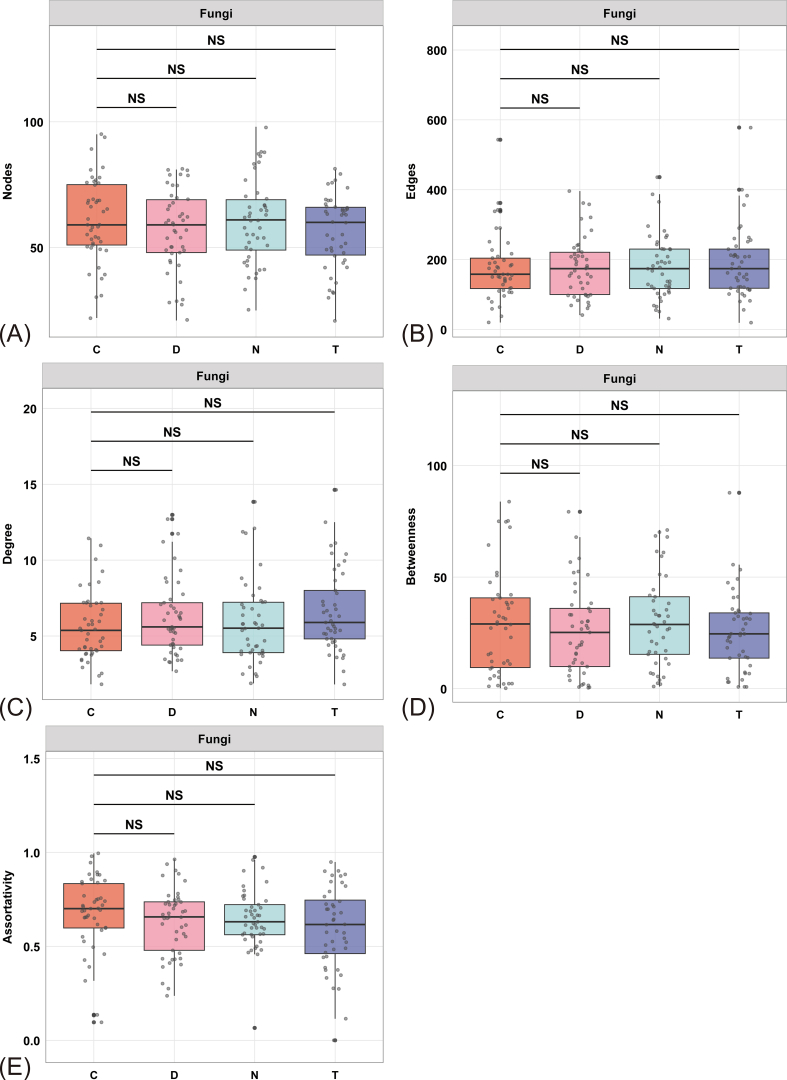


**figs5:**
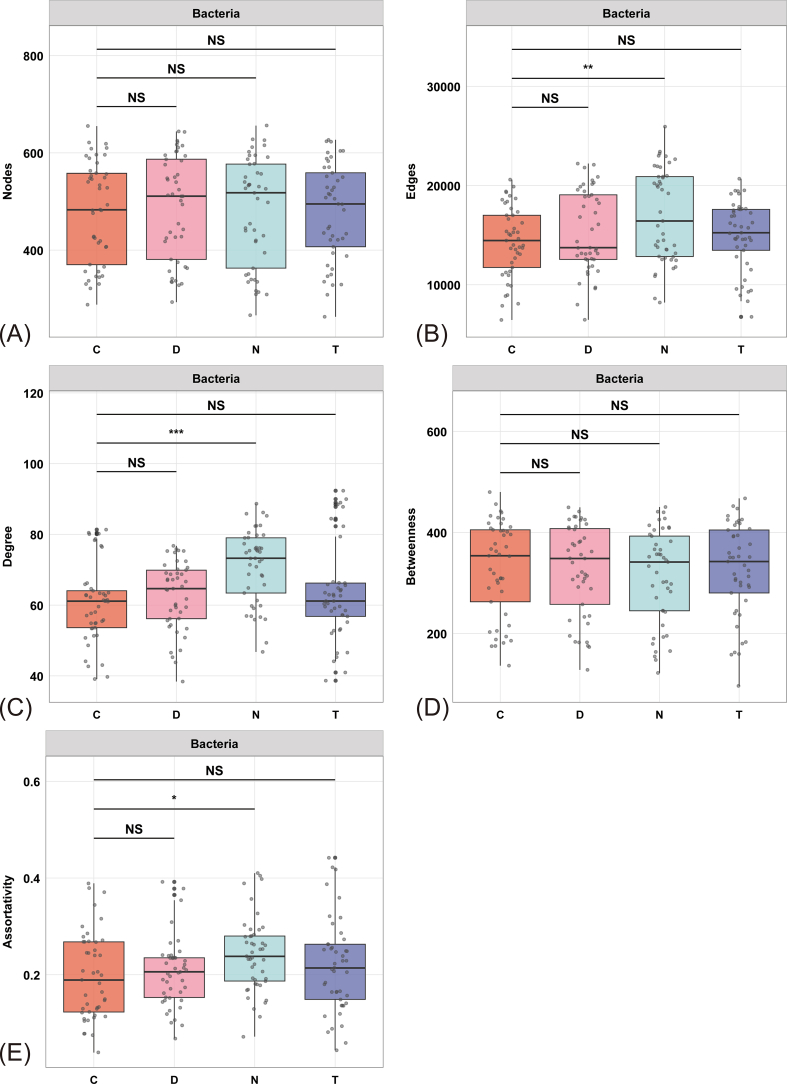


**figs6:**
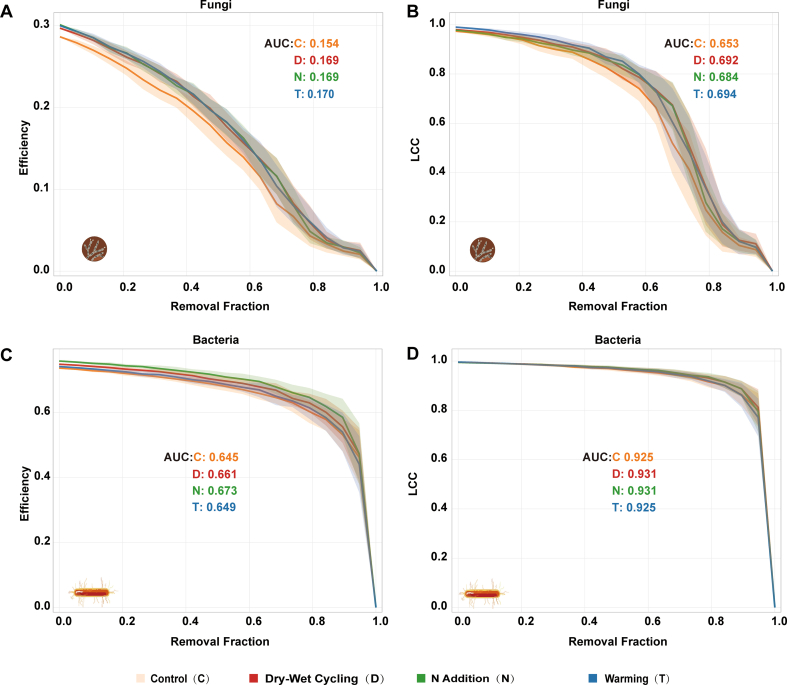


**figs7:**
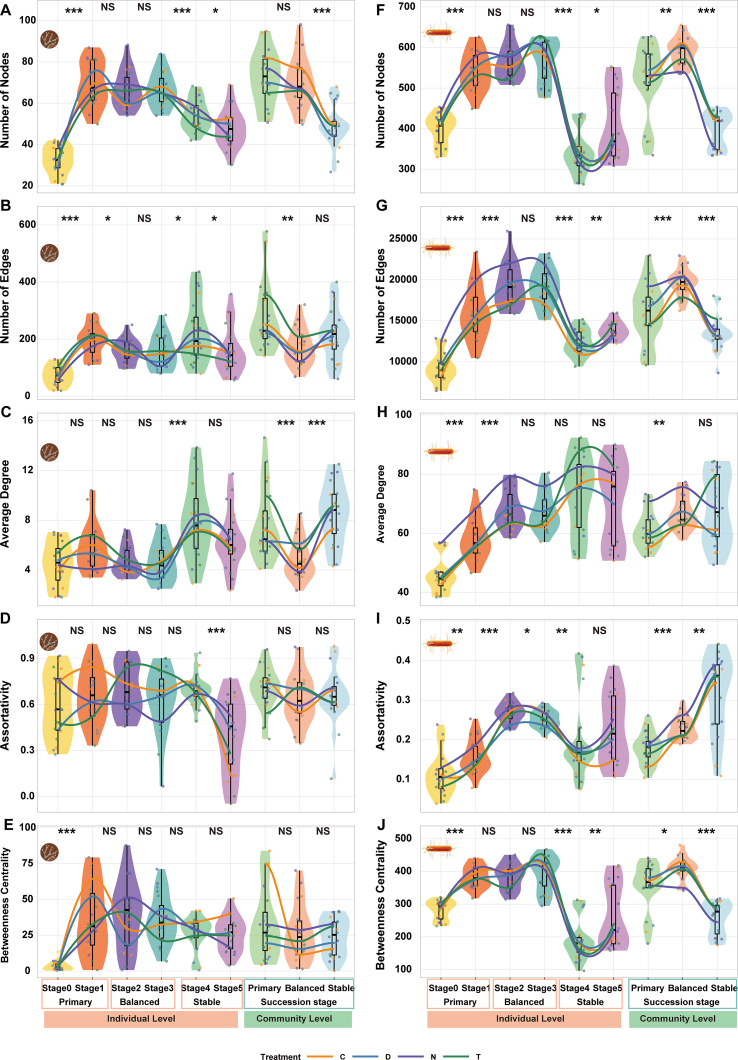


**figs8:**
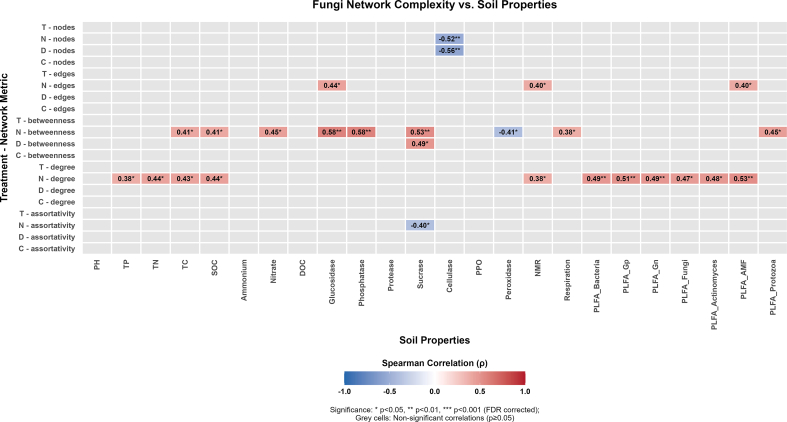


**figs9:**
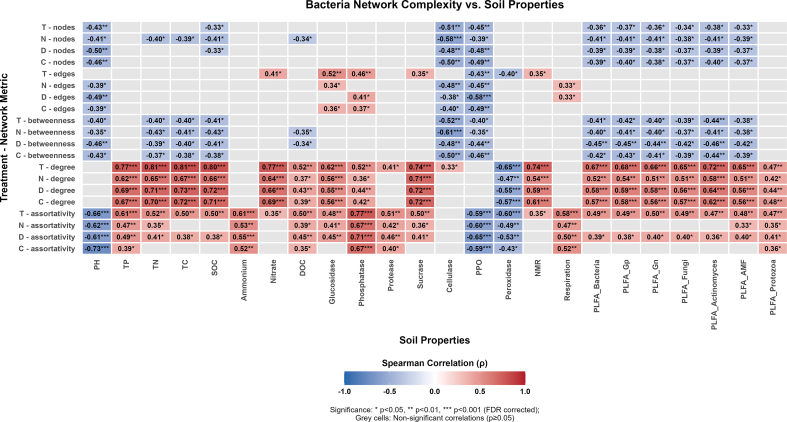


**figs10:**
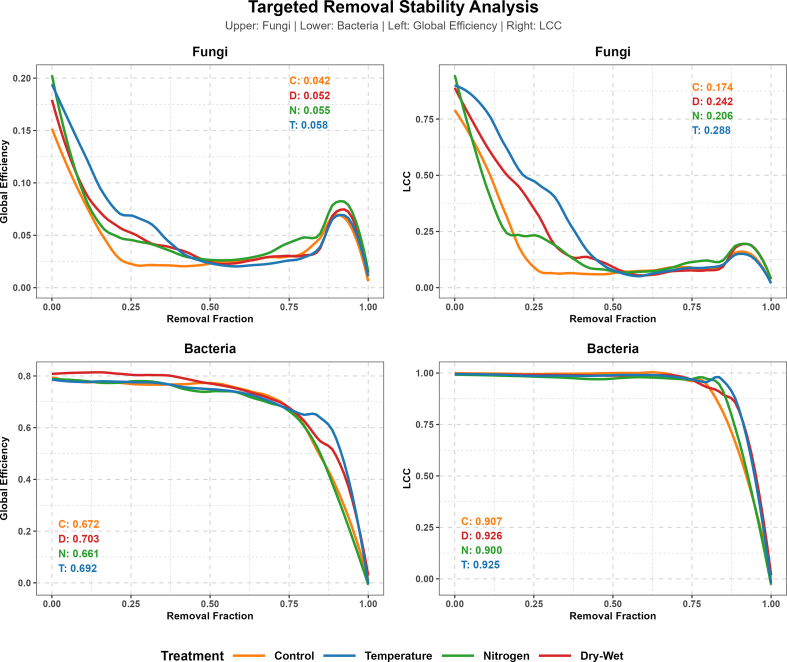


**figs11:**
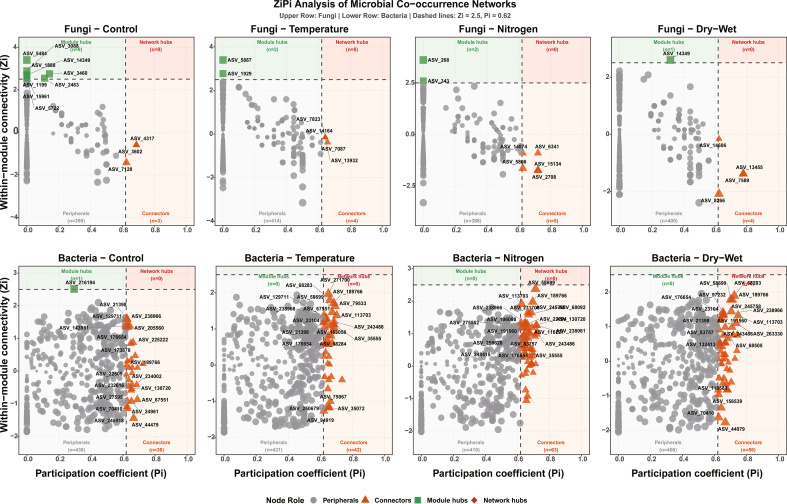


**figs12:**
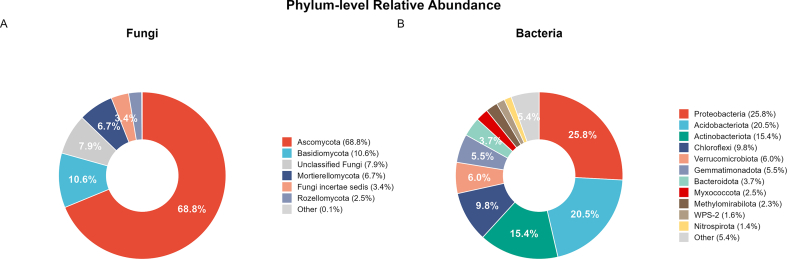


**figs13:**
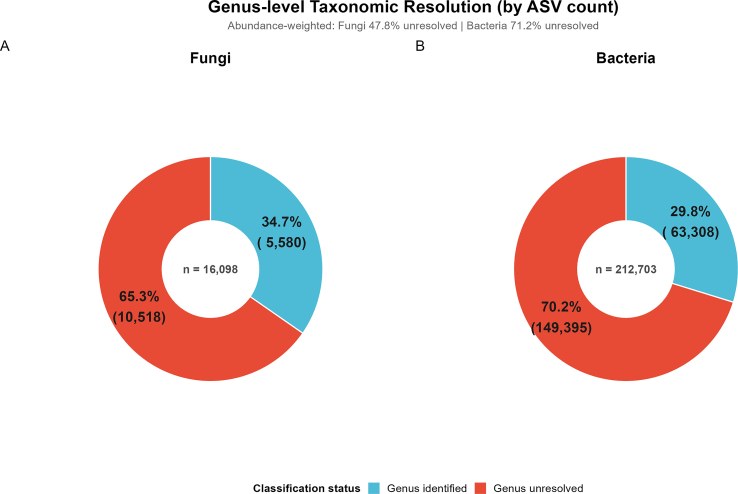


**figs14:**
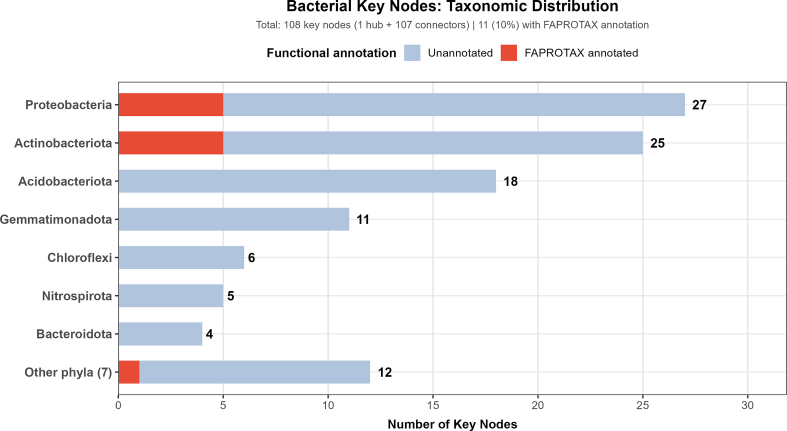


**figs15:**
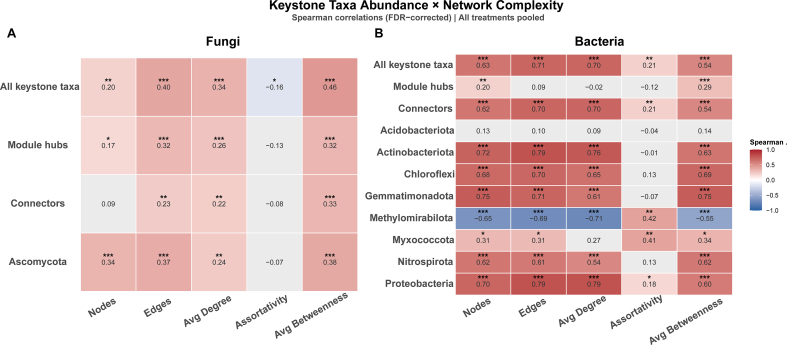


**figs16:**