# Supplementary material for: Divergent responses of microbial networks to global change scenarios during alpine cushion plant degradation
Source: Plant Divers. 2026 May 4;48(4):800–14. doi: 10.1016/j.pld.2026.04.010 (PMC13424387; doi:10.1016/j.pld.2026.04.010)

**Supporting Information 1**

**Table S1. Initial soil physicochemical properties at the community level across degradation stages (mean ± SD).**

| Indicator | Primary | Balanced | Stable | P value |
| --- | --- | --- | --- | --- |
| Ammonium (mg kg⁻¹) | 1.39 ± 0.34 | 3.78 ± 5.08 | 2.77 ± 1.23 | 0.077 |
| Cellulase (mg g⁻¹ 72 h⁻¹) | 0.2 ± 0.1 | 0.4 ± 0.2 | 0.8 ± 0.2 | 0.008 |
| DOC (g kg⁻¹) | 0.10 ± 0.02 | 0.12 ± 0.05 | 0.19 ± 0.02 | 0.009 |
| Glucosidase (mg g⁻¹ h⁻¹) | 0.5 ± 0.2 | 0.6 ± 0.3 | 0.7 ± 0.2 | 0.336 |
| NMR (mg kg⁻¹ 7 d⁻¹) | 14.35 ± 6.95 | 19.32 ± 6.29 | 32.34 ± 5.37 | 0.01 |
| Nitrate (mg kg⁻¹) | 8.11 ± 1.99 | 17.32 ± 4.01 | 30.50 ± 5.04 | 0.002 |
| pH | 5.84 ± 0.17 | 5.68 ± 0.15 | 5.82 ± 0.21 | 0.326 |
| PLFA_AMF (nmol/g) | 1.17 ± 0.40 | 0.79 ± 0.28 | 3.22 ± 1.57 | 0.005 |
| PLFA_Actinomyces (nmol/g) | 3.81 ± 0.82 | 2.56 ± 0.74 | 10.43 ± 4.58 | 0.004 |
| PLFA_Bacteria (nmol/g) | 18.64 ± 3.40 | 12.72 ± 3.59 | 58.18 ± 27.09 | 0.003 |
| PLFA_Fungi (nmol/g) | 5.80 ± 1.48 | 3.77 ± 1.48 | 12.71 ± 5.62 | 0.006 |
| PLFA_Gn (nmol/g) | 11.37 ± 1.94 | 7.86 ± 2.22 | 37.43 ± 18.20 | 0.003 |
| PLFA_Gp (nmol/g) | 7.27 ± 1.52 | 4.86 ± 1.39 | 20.76 ± 8.94 | 0.003 |
| PLFA_Protozoa (nmol/g) | 0.16 ± 0.10 | 0.16 ± 0.16 | 0.40 ± 0.31 | 0.215 |
| PPO (μg g⁻¹ 2 h⁻¹) | 191.1 ± 56.6 | 165.2 ± 28.6 | 153.3 ± 38.3 | 0.482 |
| Peroxidase (μg g⁻¹ 2 h⁻¹) | 596.0 ± 202.8 | 340.8 ± 61.9 | 228.1 ± 25.2 | 0.002 |
| Phosphatase (μg g⁻¹ 2 h⁻¹) | 256.1 ± 27.0 | 303.9 ± 76.1 | 361.0 ± 27.6 | 0.044 |
| Protease (μg g⁻¹ 24 h⁻¹) | 152.0 ± 61.6 | 176.8 ± 42.6 | 222.2 ± 38.5 | 0.121 |
| Respiration (g g⁻¹ 7 d⁻¹) | 0.07 ± 0.04 | 0.06 ± 0.03 | 0.09 ± 0.03 | 0.218 |
| SOC (g kg⁻¹) | 47.9 ± 10.0 | 60.7 ± 23.2 | 142.6 ± 50.3 | 0.008 |
| Sucrase (mg g⁻¹ 24 h⁻¹) | 27.5 ± 7.4 | 35.7 ± 17.9 | 52.4 ± 20.4 | 0.164 |
| TC (%) | 5.29 ± 1.11 | 6.53 ± 2.41 | 14.65 ± 4.87 | 0.008 |
| TN (%) | 0.45 ± 0.07 | 0.59 ± 0.18 | 1.32 ± 0.38 | 0.007 |
| TP (g kg⁻¹) | 1.02 ± 0.11 | 1.46 ± 0.20 | 2.24 ± 0.22 | 0.002 |

Values are mean ± SD (n = 5). Community level includes the Primary, Balanced, and Stable stages. P values were calculated using Kruskal–Wallis tests across stages. Abbreviations: SOC, soil organic carbon; TN, total nitrogen; TC, total carbon; TP, total phosphorus; DOC, dissolved organic carbon; NMR, nitrogen mineralization rate; PPO, polyphenol oxidase; PLFA_Bacteria, total bacterial PLFAs; PLFA_Gp, Gram-positive bacterial PLFAs; PLFA_Gn, Gram-negative bacterial PLFAs; PLFA_Fungi, fungal PLFAs; PLFA_Actinomyces, actinomycete PLFAs; PLFA_AMF, arbuscular mycorrhizal fungal PLFAs; PLFA_Protozoa, protozoan PLFAs.

**Table S2. Initial soil physicochemical properties at the individual level across degradation stages (mean ± SD).**

| Indicator | Stage 0 | Stage 1 | Stage 2 | Stage 3 | Stage 4 | Stage 5 | P value |
| --- | --- | --- | --- | --- | --- | --- | --- |
| Ammonium  (mg kg⁻¹) | 1.19 ± 0.36 | 1.58 ± 0.38 | 1.90 ± 0.60 | 5.58 ± 9.42 | 11.37 ± 18.99 | 8.95 ± 10.25 | 0.13 |
| Cellulase  (mg g⁻¹ 72 h⁻¹) | 0.4 ±  0.1 | 0.1 ±  0.0 | 0.3 ±  0.1 | 0.2 ±  0.0 | 0.6 ±  0.2 | 0.4 ±  0.2 | 0.001 |
| DOC  (g kg⁻¹) | 0.09 ± 0.01 | 0.10 ± 0.01 | 0.14 ± 0.01 | 0.17 ± 0.03 | 0.28 ± 0.08 | 0.15 ± 0.01 | < 0.001 |
| Glucosidase  (mg g⁻¹ h⁻¹) | 0.1 ±  0.0 | 0.5 ±  0.2 | 0.7 ±  0.1 | 0.7 ±  0.1 | 0.7 ±  0.0 | 0.6 ±  0.1 | 0.002 |
| NMR  (mg kg⁻¹ 7 d⁻¹) | 0.88 ± 0.81 | 10.69 ± 6.02 | 16.74 ± 4.08 | 17.83 ± 5.81 | 28.74 ± 9.78 | 21.29 ± 9.32 | 0.002 |
| Nitrate  (mg kg⁻¹) | 2.23 ± 0.94 | 10.38 ± 4.26 | 19.89 ± 7.86 | 16.06 ± 8.33 | 27.94 ± 11.65 | 15.23 ± 11.18 | 0.005 |
| pH | 6.06 ± 0.17 | 6.05 ± 0.19 | 5.35 ± 0.09 | 5.67 ± 0.26 | 6.03 ± 0.79 | 6.14 ± 0.29 | 0.027 |
| PLFA_AMF  (nmol/g) | 0.23 ± 0.05 | 0.85 ± 0.31 | 1.76 ± 0.52 | 1.64 ± 0.22 | 4.16 ± 0.68 | 2.59 ± 0.85 | < 0.001 |
| PLFA_Actinomyces  (nmol/g) | 0.91 ± 0.22 | 2.79 ± 0.83 | 6.21 ± 1.67 | 5.28 ± 0.80 | 11.68 ± 1.51 | 8.02 ± 2.83 | < 0.001 |
| PLFA_Bacteria  (nmol/g) | 4.48 ± 0.91 | 14.55 ± 4.96 | 33.06 ± 10.18 | 29.26 ± 5.32 | 67.96 ± 13.01 | 43.41 ± 12.73 | < 0.001 |
| PLFA_Fungi  (nmol/g) | 1.12 ± 0.18 | 4.23 ± 1.58 | 8.02 ± 2.19 | 7.36 ± 0.70 | 16.74 ± 3.76 | 10.55 ± 3.43 | < 0.001 |
| PLFA_Gn  (nmol/g) | 2.57 ± 0.75 | 8.57 ± 3.04 | 20.94 ± 6.98 | 18.57 ± 3.58 | 45.16 ± 9.71 | 27.79 ± 8.48 | < 0.001 |
| PLFA_Gp  (nmol/g) | 1.90 ± 0.39 | 5.97 ± 1.93 | 12.12 ± 3.20 | 10.69 ± 1.76 | 22.80 ± 3.69 | 15.62 ± 4.29 | < 0.001 |
| PLFA_Protozoa  (nmol/g) | 0.00 ± 0.00 | 0.15 ± 0.12 | 0.32 ± 0.21 | 0.37 ± 0.05 | 0.58 ± 0.25 | 0.32 ± 0.19 | 0.006 |
| PPO  (μg g⁻¹ 2 h⁻¹) | 289.7 ± 60.1 | 194.6 ± 17.7 | 149.8 ± 60.6 | 150.7 ± 19.4 | 231.9 ± 35.8 | 236.3 ± 75.7 | 0.004 |
| Peroxidase  (μg g⁻¹ 2 h⁻¹) | 2089.8 ± 258.7 | 1246.3 ± 333.0 | 435.1 ± 93.0 | 450.3 ± 216.4 | 441.8 ± 159.8 | 365.1 ± 63.0 | 0.001 |
| Phosphatase  (μg g⁻¹ 2 h⁻¹) | 74.1 ± 18.9 | 201.0 ± 57.7 | 365.0 ± 55.5 | 332.9 ± 48.8 | 296.5 ± 55.2 | 258.2 ± 45.3 | < 0.001 |
| Protease  (μg g⁻¹ 24 h⁻¹) | 47.3 ± 12.4 | 158.3 ± 65.4 | 175.4 ± 33.2 | 200.7 ± 41.9 | 214.9 ± 34.4 | 202.1 ± 21.3 | 0.008 |
| Respiration  (g g⁻¹ 7 d⁻¹) | 0.04 ± 0.02 | 0.06 ± 0.03 | 0.14 ± 0.03 | 0.14 ± 0.03 | 0.12 ± 0.06 | 0.08 ± 0.05 | 0.005 |
| SOC  (g kg⁻¹) | 12.1 ± 3.3 | 36.1 ± 15.5 | 88.0 ± 18.4 | 67.6 ± 13.4 | 131.7 ± 23.0 | 105.2 ± 48.8 | < 0.001 |
| Sucrase  (mg g⁻¹ 24 h⁻¹) | 4.1 ± 2.1 | 25.6 ± 11.9 | 59.0 ± 16.3 | 55.7 ± 9.3 | 61.7 ± 10.1 | 58.2 ± 23.5 | 0.002 |
| TC  (%) | 1.25 ± 0.34 | 3.76 ± 1.58 | 9.20 ± 1.75 | 7.62 ± 1.53 | 13.54 ± 2.30 | 11.01 ± 4.95 | < 0.001 |
| TN  (%) | 0.15 ± 0.03 | 0.34 ± 0.12 | 0.83 ± 0.15 | 0.67 ± 0.15 | 1.13 ± 0.18 | 0.98 ± 0.40 | < 0.001 |
| TP  (g kg⁻¹) | 0.74 ± 0.13 | 0.85 ± 0.17 | 1.94 ± 0.33 | 1.69 ± 0.45 | 1.79 ± 0.47 | 2.02 ± 0.47 | 0.001 |

Values are mean ± SD (n = 5). Individual level includes Stage 0 (bare land), Stage 1 (healthy cushion with 0% beneficiary cover), Stage 2 (approximately 20% beneficiary cover), Stage 3 (approximately 50% beneficiary cover), Stage 4 (approximately 80% beneficiary cover), and Stage 5 (cushion senescence, approximately 100% beneficiary cover). P values were calculated using Kruskal–Wallis tests across stages. Abbreviations are the same as in Table S1.

**Table S3. Network robustness under random and targeted node removal, and topological role distribution.**

|  | Robustness (AUC) | | | | | | | Topological roles | | | |
| --- | --- | --- | --- | --- | --- | --- | --- | --- | --- | --- | --- |
| Taxa | Treat | LCC_R | LCC_T | Δ LCC | GE_R | GE_T | Δ GE | Peri | Conn | M-hub | N-hub |
| Fungi | C | 0.355 | 0.174 | -51% | 0.062 | 0.042 | -32% | 390 | 3 | 9 | 0 |
| Fungi | T | 0.434 | 0.288 | -34% | 0.080 | 0.058 | -28% | 414 | 4 | 2 | 0 |
| Fungi | N | 0.433 | 0.206 | -52% | 0.085 | 0.055 | -35% | 386 | 5 | 2 | 0 |
| Fungi | D | 0.410 | 0.242 | -41% | 0.074 | 0.052 | -29% | 400 | 4 | 1 | 0 |
| Bacteria | C | 0.931 | 0.907 | -3% | 0.703 | 0.672 | -4% | 436 | 30 | 1 | 0 |
| Bacteria | T | 0.922 | 0.925 | +0.3% | 0.690 | 0.692 | +0.2% | 431 | 42 | 0 | 0 |
| Bacteria | N | 0.916 | 0.900 | -2% | 0.691 | 0.661 | -4% | 410 | 63 | 0 | 0 |
| Bacteria | D | 0.935 | 0.926 | -1% | 0.723 | 0.703 | -3% | 406 | 56 | 0 | 0 |

Abbreviations: Treat, treatment; LCC_R, LCC-AUC under random removal; LCC_T, LCC-AUC under targeted removal; ΔLCC, relative change in LCC-AUC from random to targeted removal; GE_R, GE-AUC under random removal; GE_T, GE-AUC under targeted removal; ΔGE, relative change in GE-AUC from random to targeted removal; Peri, peripherals; Conn, connectors; M-hub, module hubs; N-hub, network hubs. LCC, largest connected component; GE, global efficiency; AUC, area under the curve. Node roles were classified based on within-module connectivity (Zi) and among-module connectivity (Pi) (Guimerà and Amaral, 2005): peripherals (Zi ≤ 2.5, Pi ≤ 0.62), connectors (Zi ≤ 2.5, Pi > 0.62), module hubs (Zi > 2.5, Pi ≤ 0.62), and network hubs (Zi > 2.5, Pi > 0.62).

**Table S4. Spearman correlations between keystone taxa abundance and network complexity metrics**

**A. Fungal keystone taxa correlations (all samples pooled, *n* = 180)**

| Keystone variable | Nodes | Edges | Avg Degree | Assortativity | Avg Betweenness |
| --- | --- | --- | --- | --- | --- |
| Total keystone | 0.205** | 0.398*** | 0.344*** | -0.157* | 0.460*** |
| Module hubs | 0.172* | 0.317*** | 0.265*** | -0.132 ns | 0.324*** |
| Connectors | 0.092 ns | 0.233** | 0.222** | -0.081 ns | 0.328*** |
| Ascomycota | 0.335*** | 0.372*** | 0.238** | -0.071 ns | 0.383*** |

**B. Bacterial keystone taxa correlations (all samples pooled, *n* = 180)**

| Keystone variable | Nodes | Edges | Avg Degree | Assortativity | Avg Betweenness |
| --- | --- | --- | --- | --- | --- |
| Total keystone | 0.630*** | 0.706*** | 0.701*** | 0.208** | 0.544*** |
| Module hubs | 0.205** | 0.087 ns | -0.023 ns | -0.124 ns | 0.288*** |
| Connectors | 0.624*** | 0.701*** | 0.698*** | 0.209** | 0.538*** |
| By phylum: |  |  |  |  |  |
| Actinobacteriota | 0.722*** | 0.791*** | 0.762*** | -0.015 ns | 0.633*** |
| Proteobacteria | 0.704*** | 0.792*** | 0.790*** | 0.177* | 0.599*** |
| Gemmatimonadota | 0.751*** | 0.709*** | 0.611*** | -0.072 ns | 0.746*** |
| Acidobacteriota | 0.132 ns | 0.104 ns | 0.087 ns | -0.045 ns | 0.141 ns |
| Methylomirabilota | -0.649*** | -0.685*** | -0.715*** | 0.417** | -0.546*** |
| Nitrospirota | 0.619*** | 0.609*** | 0.540*** | 0.130 ns | 0.615*** |
| Chloroflexi | 0.683*** | 0.695*** | 0.648*** | 0.129 ns | 0.694*** |
| Myxococcota | 0.315* | 0.312* | 0.269 ns | 0.411** | 0.337* |

Spearman rank correlations (*ρ*) between keystone taxa relative abundance and network complexity metrics. Keystone taxa were defined as module hubs (Zi > 2.5) and connectors (Pi > 0.62) based on the Zi-Pi classification. All analyses were performed on pooled samples across four treatments (*n* = 180). Significance levels: **P* < 0.05, ***P* < 0.01, ****P* < 0.001 after FDR correction; ns, not significant.

**Fig. S1. Cumulative abundance distribution of ASVs.** The curves show the cumulative percentage of total sequences as a function of ASV rank (sorted by abundance). Vertical dashed lines indicate the top 500 (red) and top 1,000 (blue) ASVs. For fungi, the top 500 and 1,000 ASVs represented 70.7% and 80.4% of total sequences, respectively. For bacteria, the top 500 and 1,000 ASVs represented 38.3% and 50.4% of total sequences, respectively.

**

**

**Fig. S2.** To validate the robustness of our approach, we conducted a verification analysis using the 1,000 most prevalent ASVs, generating the subsequent fungal co-occurrence networks presented below.

(A) Control, (B) Dry-wet cycling, (C) Nitrogen addition, (D) Warming.


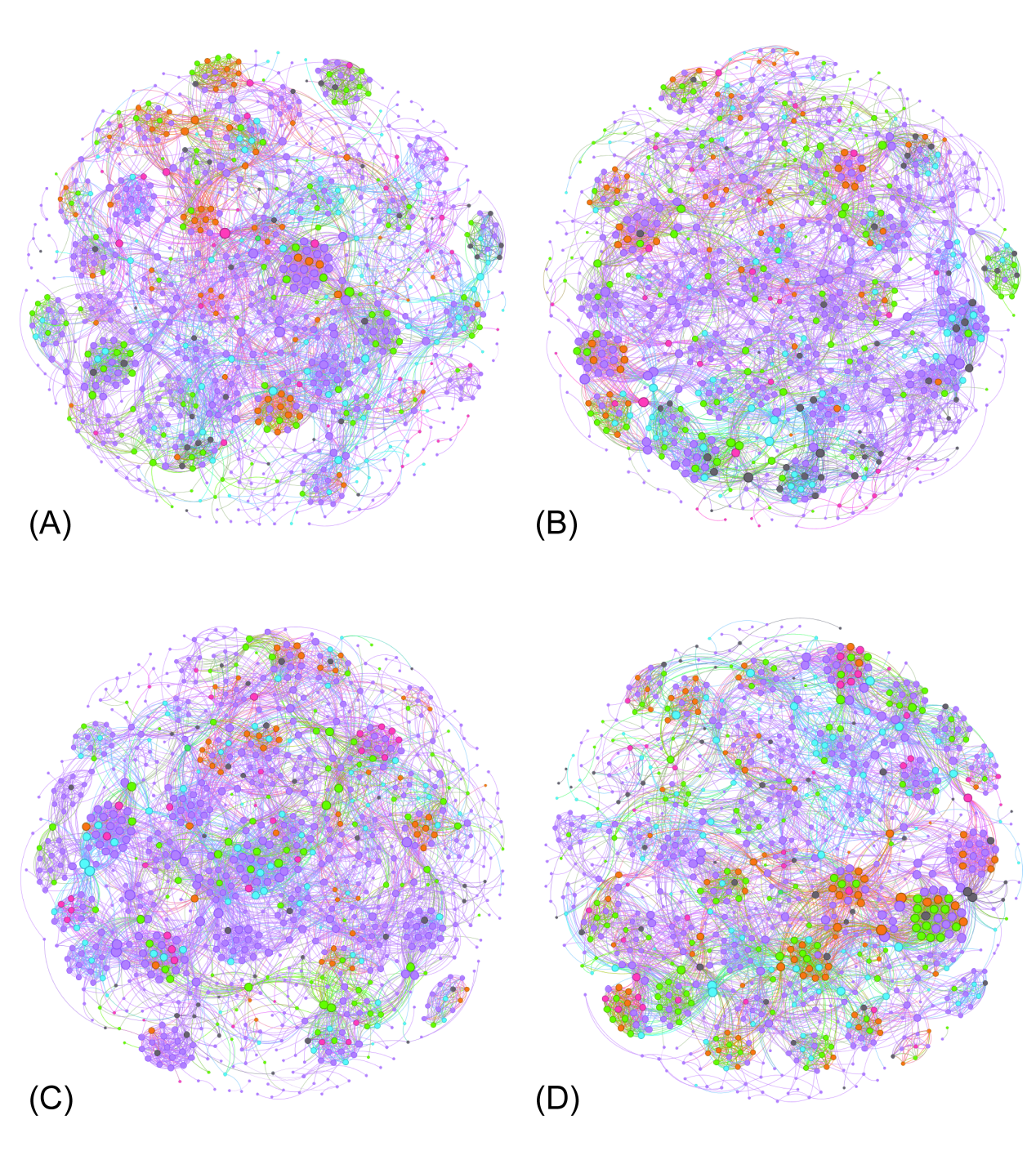


**Fig. S3.** To validate the robustness of our approach, we conducted a verification analysis using the 1,000 most prevalent ASVs, generating the subsequent bacterial co-occurrence networks presented below.

(A): Control, (B): Dry-wet cycling, (C): Nitrogen addition, (D): Warming.


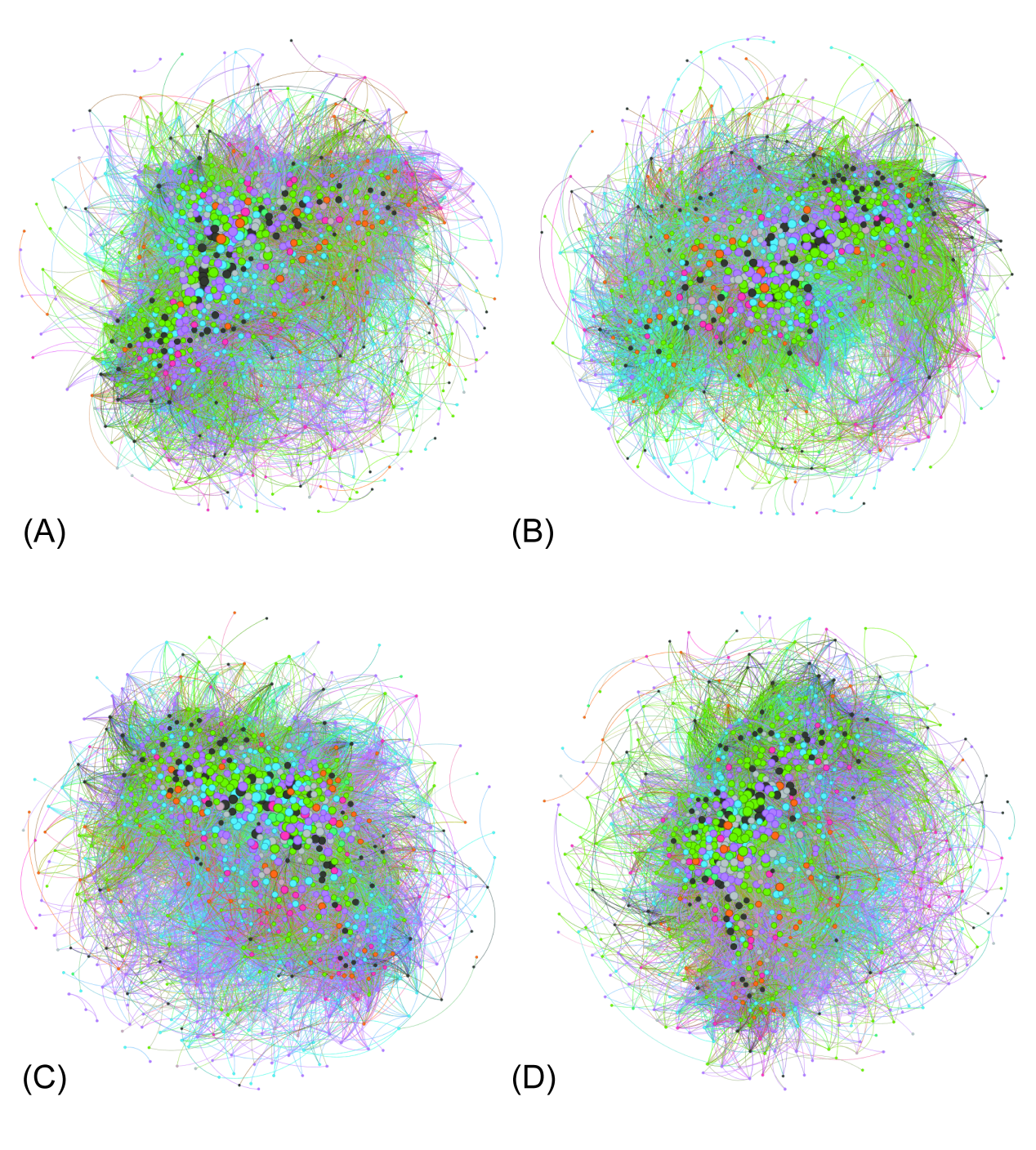


**Fig. S4.** To validate the robustness of our approach, we performed a verification analysis using the 1,000 most prevalent ASVs, generating the subsequent boxplots illustrating fungal complexity metrics presented below.

(A): nodes, (B): edges, (C): degree, (D): betweenness, (E): assortativity.


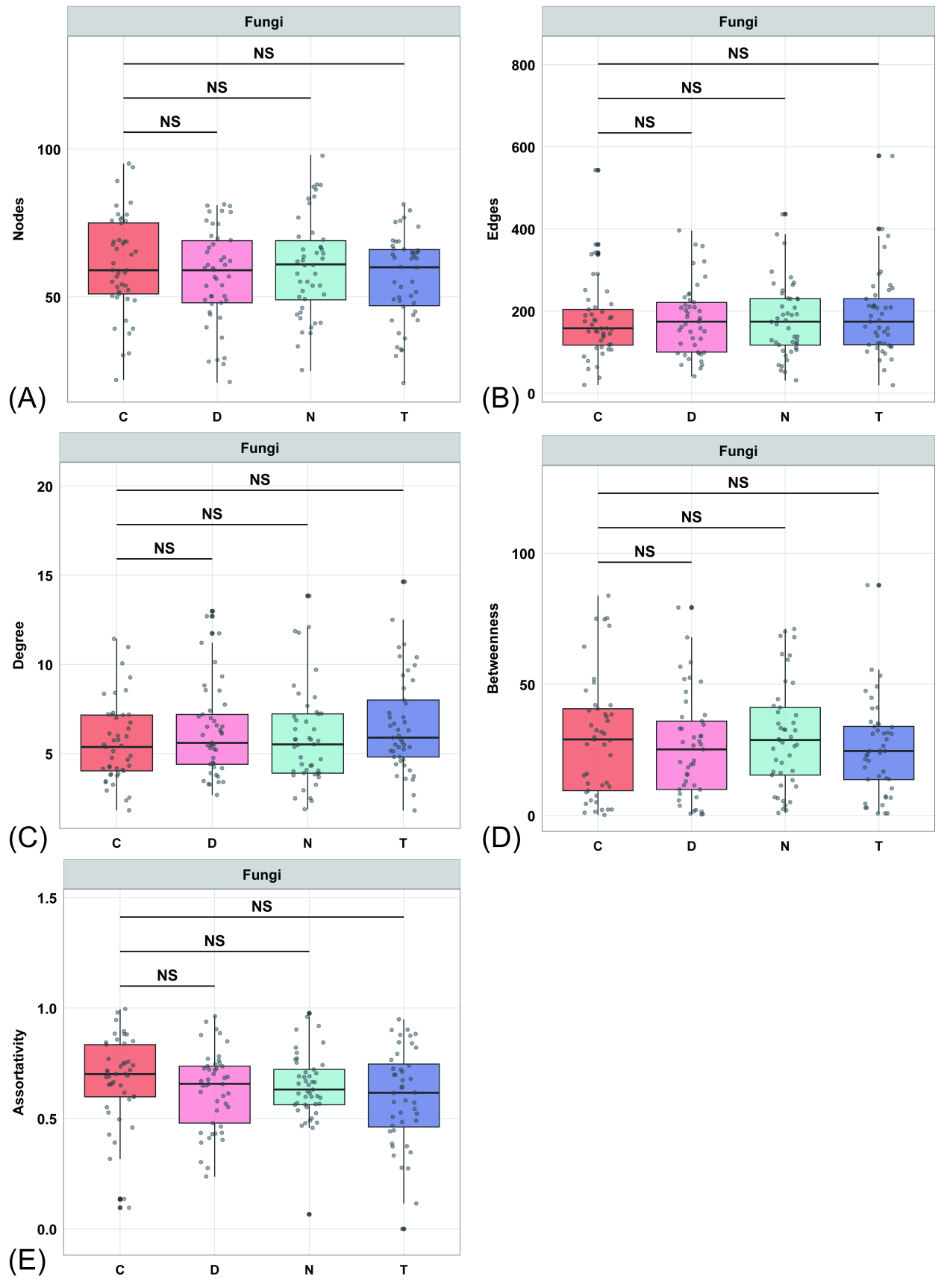


**Fig. S5.** To validate the robustness of our approach, we conducted a verification analysis using the 1,000 most prevalent ASVs, generating the subsequent boxplots of bacterial complexity metrics presented below.

(A): nodes, (B): edges, (C): degree, (D): betweenness, (E): assortativity.


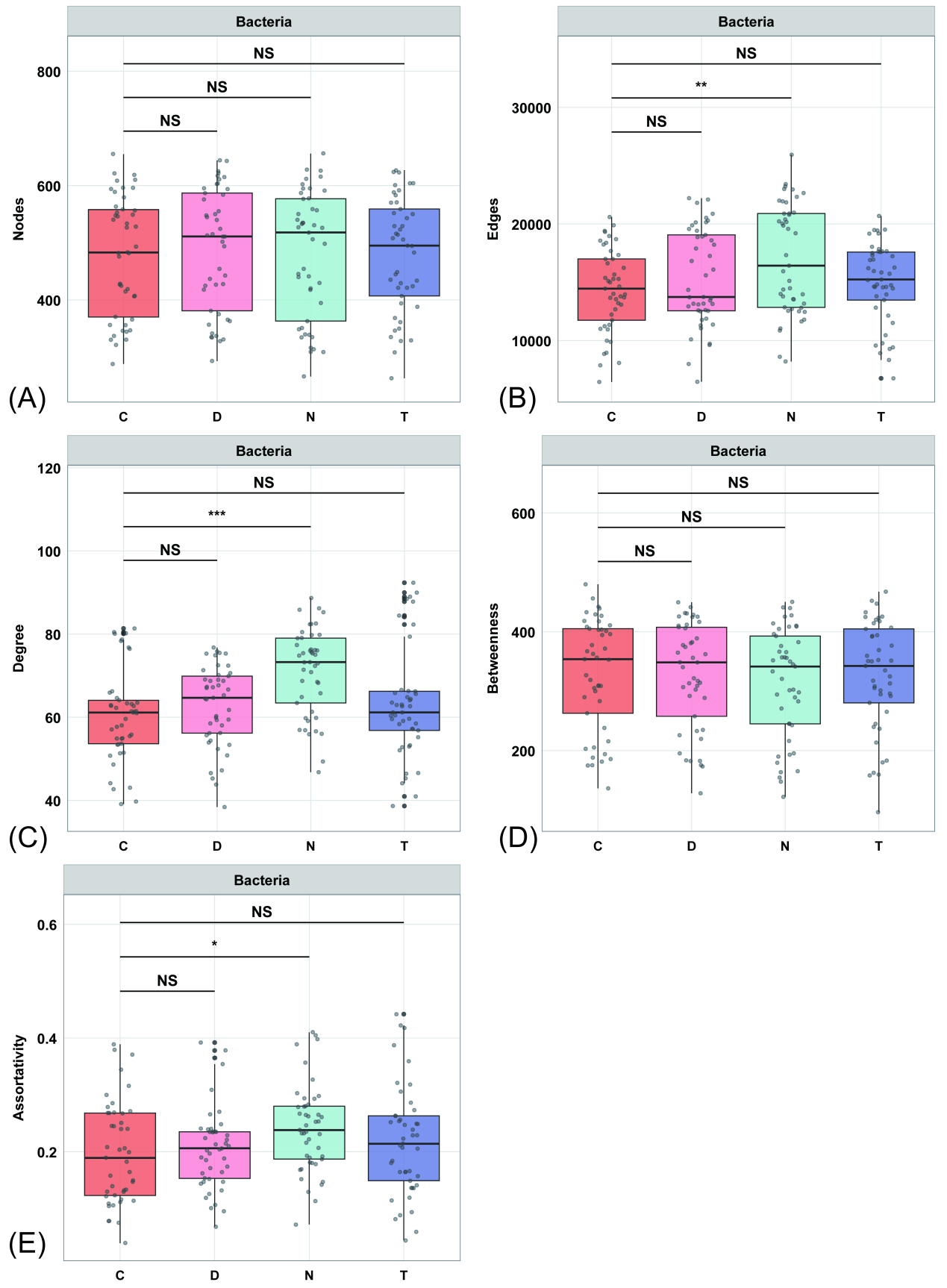


**Fig. S6.** To validate the robustness of our approach, we conducted a verification analysis using the top 1,000 ASVs by abundance, yielding the following network stability profiles.

A: Fungal global efficiency(GE), B: Fungal largest connected component (LCC),

C: Bacterial global efficiency(GE), D: Bacterial largest connected component (LCC). AUC: the area under the curve


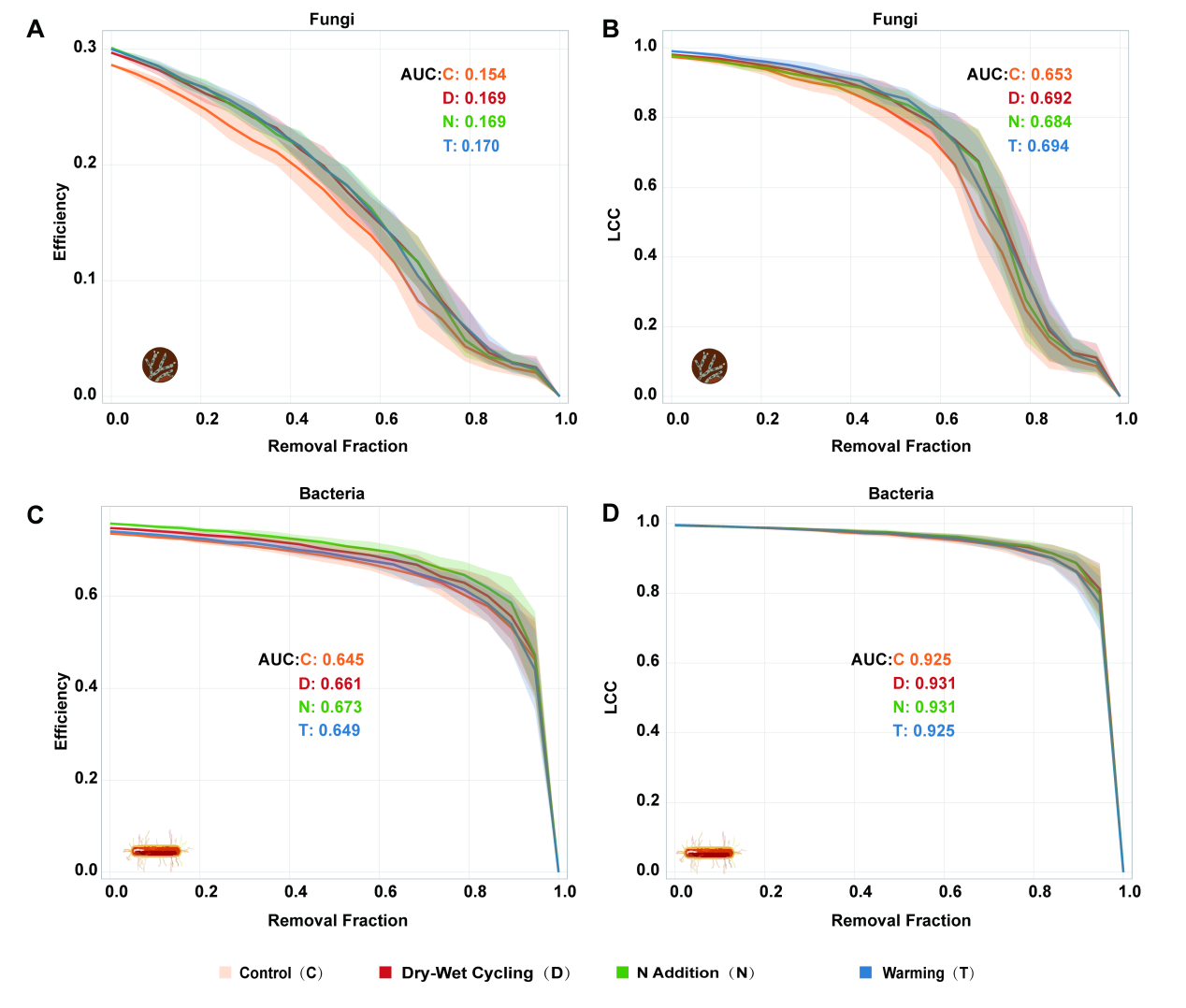


**Fig. S7.** To validate the robustness of our approach, we performed a verification analysis using the 1,000 most prevalent ASVs, generating the hybrid box-violin plots below that visualize network complexity metrics. Panels A-E display fungal metrics, and panels F-J display bacterial metrics, with specific indicators as shown in the figure.


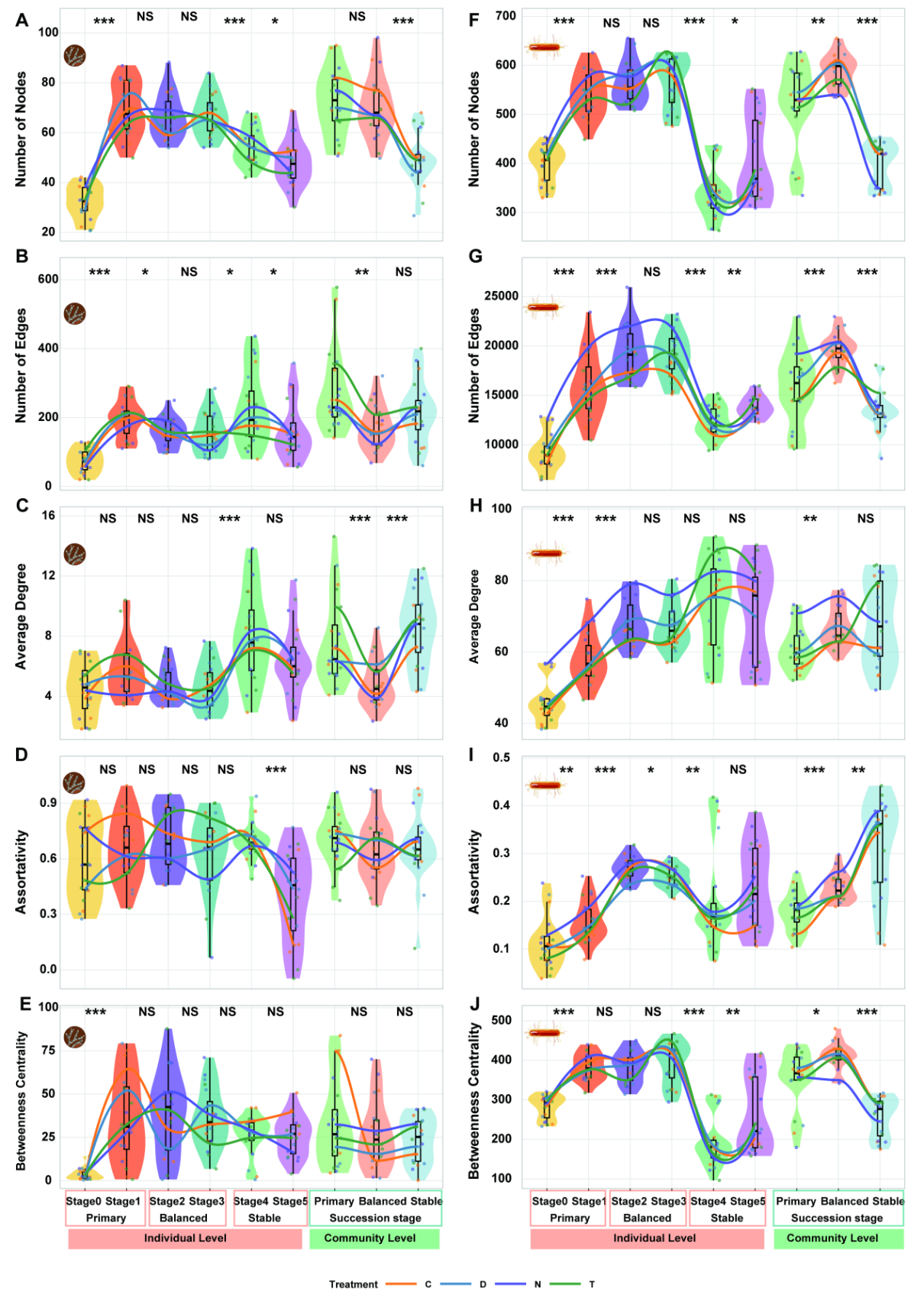


**Fig. S8.** To validate the robustness of our approach, we performed a verification analysis using the 1,000 most prevalent ASVs, generating the following heatmap of fungal complexity metrics versus initial soil properties.

A
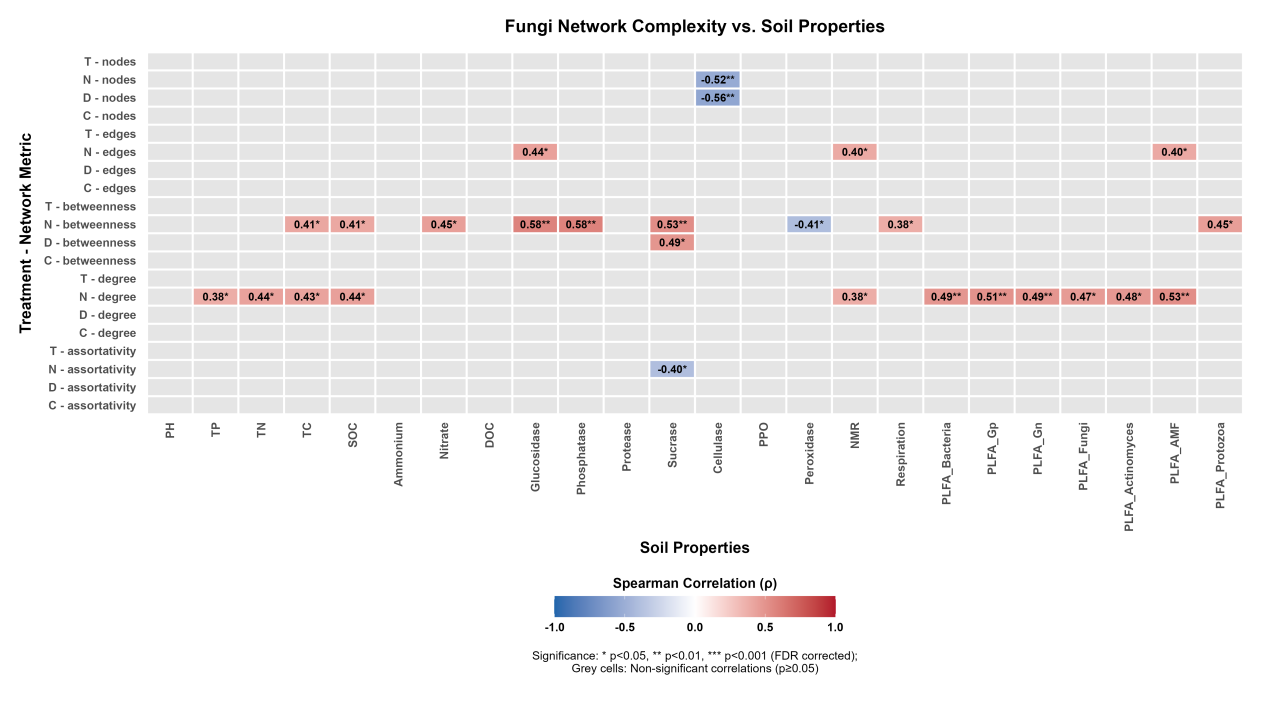


**Fig. S9.** To validate the robustness of our approach, we performed a verification analysis using the 1,000 most prevalent ASVs, generating the following heatmap of bacterial complexity metrics versus initial soil properties.

A
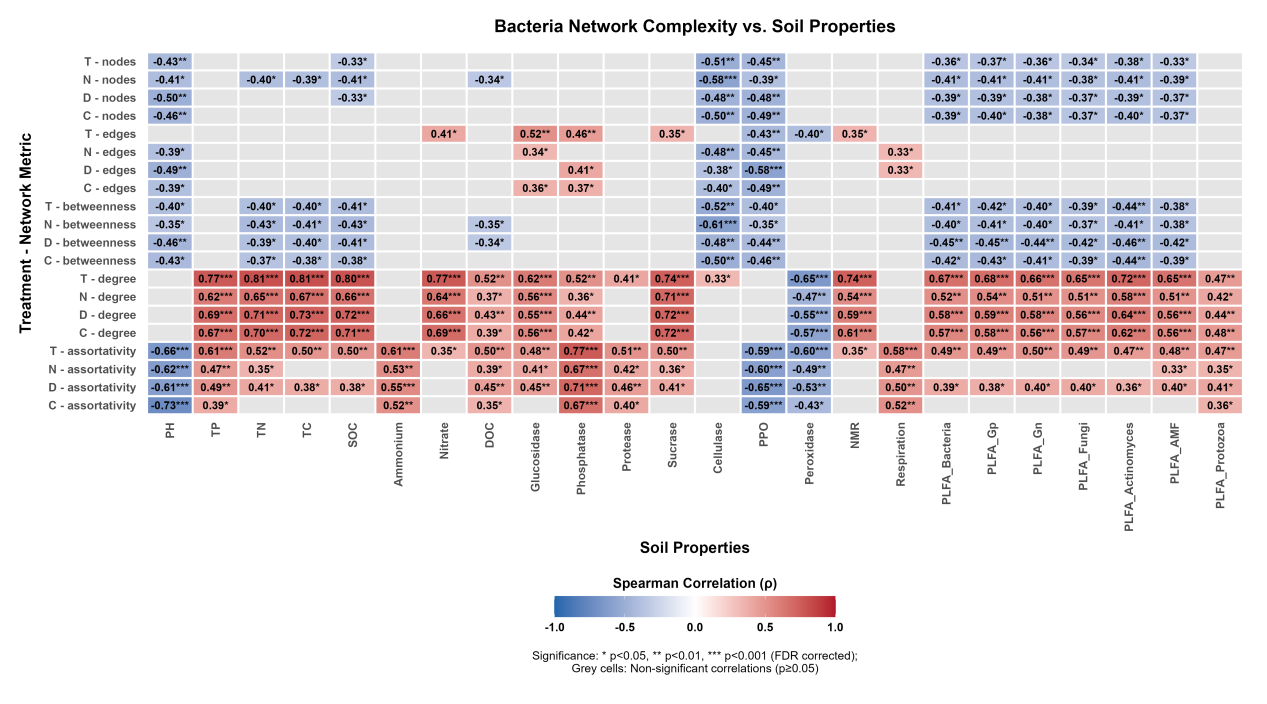


**Fig. S10. Targeted removal stability analysis of microbial co-occurrence networks.** Nodes were removed in descending order of topological importance (|Zi| + Pi). Upper row: fungal networks; Lower row: bacterial networks; Left column: global efficiency; Right column: proportion of nodes remaining in the largest connected component (LCC). Lines represent different treatments: Control (orange), Warming (blue), Nitrogen addition (green), and Dry-wet cycling (red). Values shown (C, T, N, D) indicate the area under the curve (AUC) for each treatment. Fungal networks collapsed rapidly under targeted removal (LCC-AUC: 0.174–0.288; GE-AUC: 0.042–0.058), whereas bacterial networks maintained high connectivity throughout the removal process (LCC-AUC: 0.900–0.926; GE-AUC: 0.661–0.703).


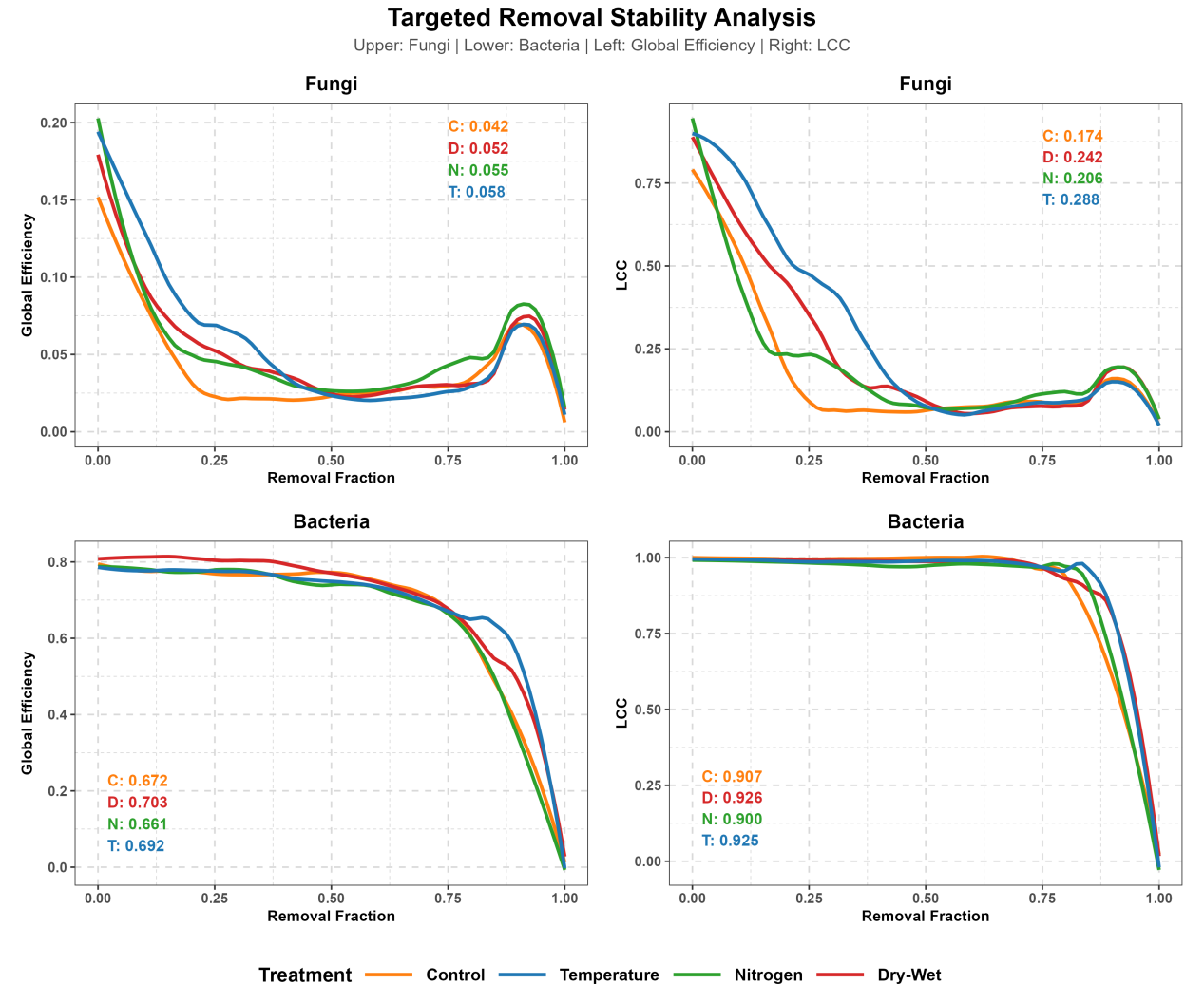


**Fig. S11. Zi-Pi plots showing topological roles of nodes in fungal (top row) and bacterial (bottom row) co-occurrence networks under different global change scenarios.** Nodes were classified into four categories based on within-module connectivity (Zi) and among-module connectivity (Pi) (Guimerà and Amaral, 2005): peripherals (Zi ≤ 2.5, Pi ≤ 0.62; gray circles), connectors (Zi ≤ 2.5, Pi > 0.62; orange triangles), module hubs (Zi > 2.5, Pi ≤ 0.62; green squares), and network hubs (Zi > 2.5, Pi > 0.62; red diamonds). Dashed lines indicate the thresholds (Zi = 2.5, Pi = 0.62). Fungal networks contained more module hubs (1–9) but fewer connectors (3–5), while bacterial networks contained almost no module hubs but possessed numerous connectors (30–63), explaining their contrasting vulnerability to targeted node removal.


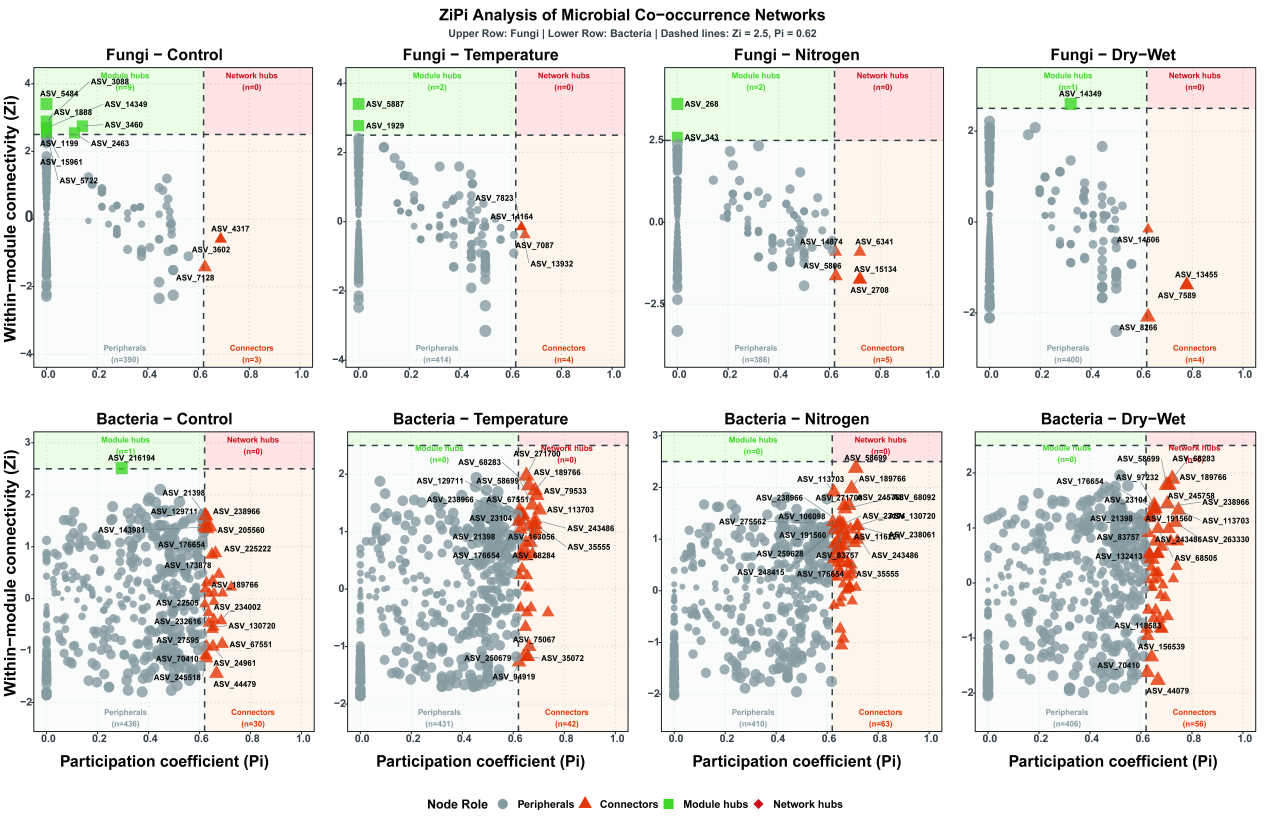


**Fig. S12. Phylum-level taxonomic composition of fungal and bacterial communities.** Phylum-level relative abundance of fungal (A) and bacterial (B) communities across all samples. For fungi, Ascomycota dominated the community (68.8%), followed by Basidiomycota (10.6%) and unclassified fungi (7.9%). For bacteria, Proteobacteria was the most abundant phylum (25.8%), followed by Acidobacteriota (20.5%) and Actinobacteriota (15.4%). Phyla with relative abundance < 2% were grouped into “Other”. Data represent mean relative abundance across all treatments (*n* = 180).


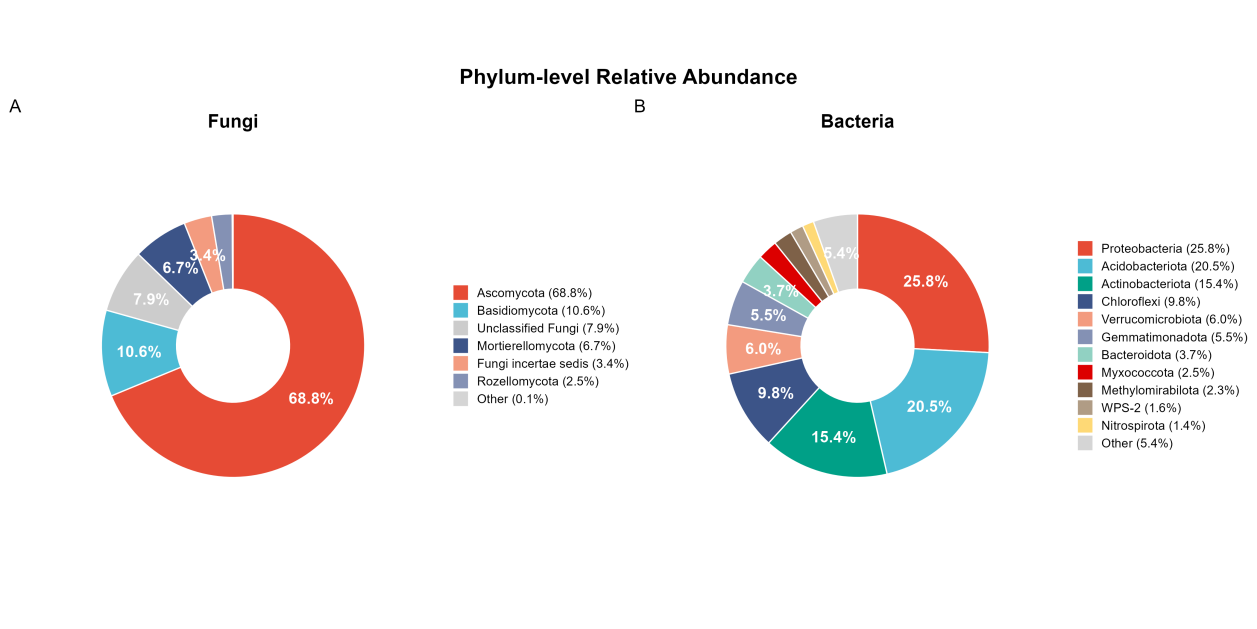


**Fig. S13. Genus-level taxonomic resolution of fungal and bacterial ASVs.** Genus-level taxonomic resolution for fungal (A) and bacterial (B) communities based on ASV counts. Donut charts show the proportion of ASVs that could be classified to the genus level (blue) versus those that remained unresolved (red). For fungi, 34.7% of ASVs (5,580 of 16,098) were classified to genus level. For bacteria, only 29.8% of ASVs (63,308 of 212,703) achieved genus-level classification, indicating substantially higher taxonomic novelty. Abundance-weighted unresolved rates were 47.8% for fungi and 71.2% for bacteria, reflecting that many abundant bacterial ASVs belong to undescribed genera.


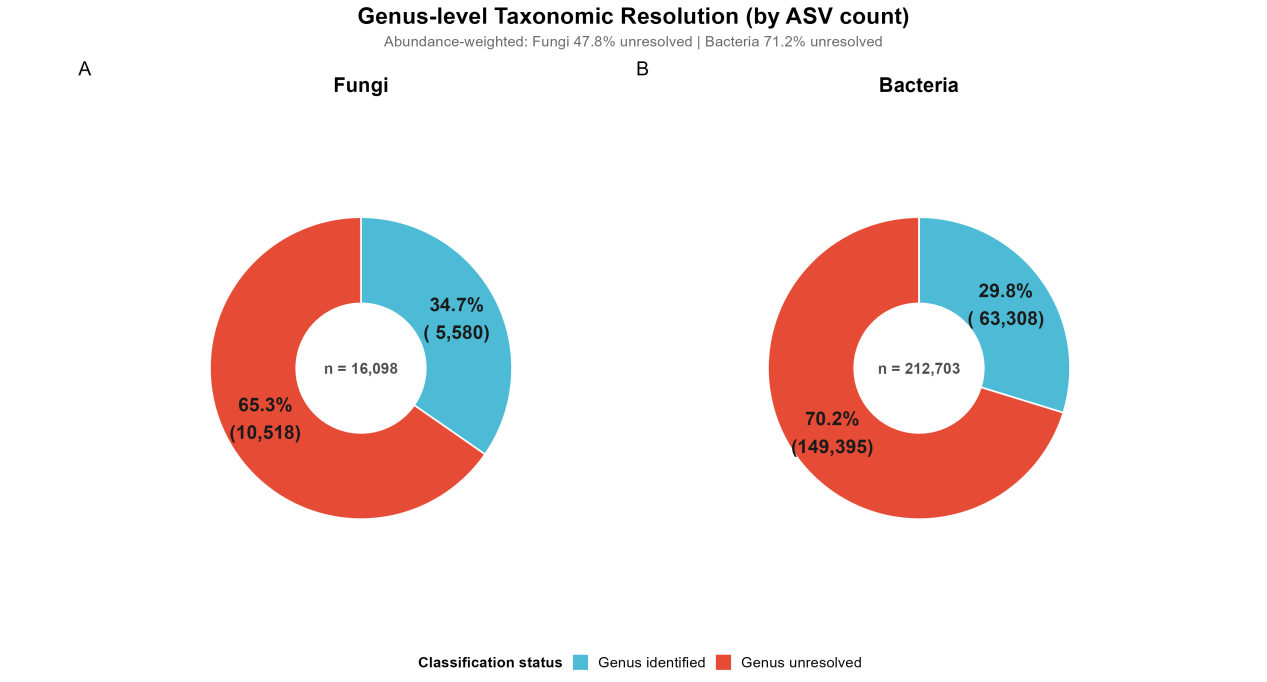


**Fig. S14. Taxonomic distribution of bacterial keystone nodes**. Taxonomic distribution of bacterial keystone nodes across phyla. A total of 108 keystone nodes were identified, comprising 1 module hub and 107 connectors. Proteobacteria (*n* = 27) and Actinobacteriota (*n* = 25) harbored the most keystone nodes, followed by Acidobacteriota (*n* = 18) and Gemmatimonadota (*n* = 11). Bar colors indicate functional annotation status: only 11 nodes (10%) could be annotated using the FAPROTAX database, highlighting the predominance of functionally uncharacterized taxa among bacterial keystones. “Other phyla” includes 7 additional phyla with ≤ 4 keystone nodes each. Note: Fungal networks contained only 24 keystone nodes (13 module hubs, 11 connectors, predominantly Ascomycota), reflecting their hub-dependent structure; phylum-level distribution is not shown due to small sample size.


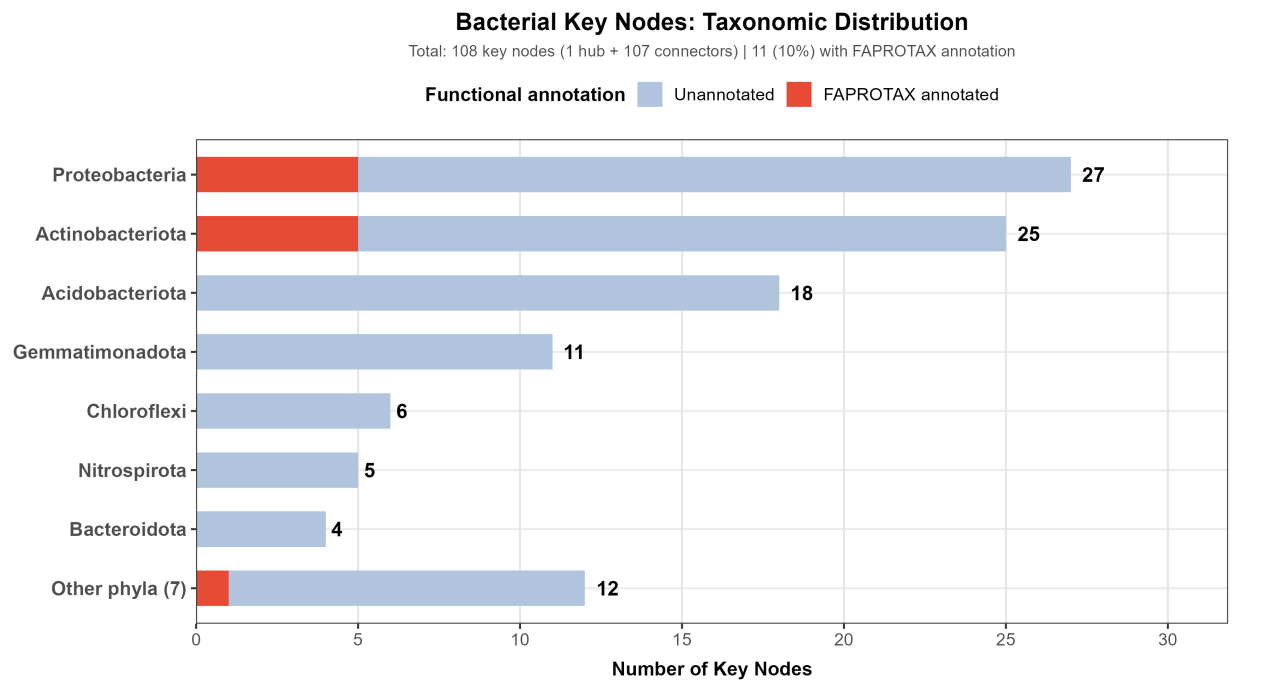


**Fig. S15. Heatmap of correlations between keystone taxa abundance and network complexity metrics.** Spearman correlation heatmap showing relationships between keystone taxa relative abundance and network complexity metrics for fungi (A) and bacteria (B). Data were pooled across all treatments (*n* = 180). Cell colors indicate correlation coefficients (*ρ*): red = positive correlation, blue = negative correlation. Significance levels after FDR correction: **P* < 0.05, ***P* < 0.01, ****P* < 0.001. For bacteria, total keystone abundance showed strong positive correlations with Nodes (*ρ* = 0.63), Edges (*ρ* = 0.71), and Average Degree (*ρ* = 0.70). Actinobacteriota and Proteobacteria exhibited the strongest positive associations, while Methylomirabilota showed consistent negative correlations. For fungi, correlations were generally weaker, with the strongest associations observed for Average Betweenness (*ρ* = 0.46).


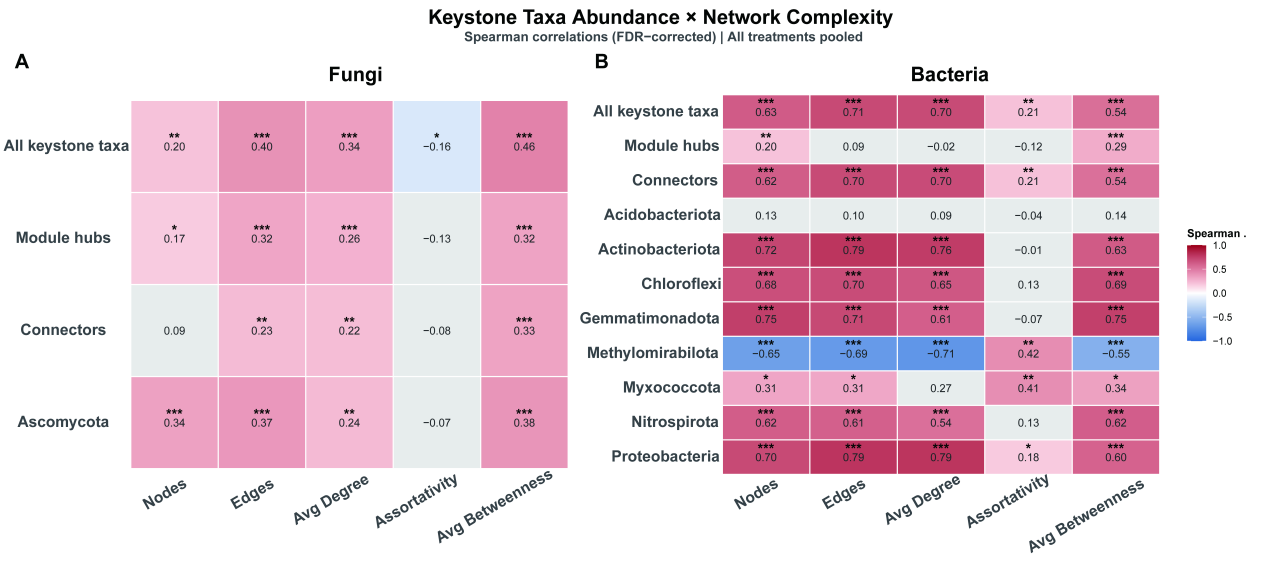


**Fig. S16. Treatment-specific correlations between keystone taxa abundance and network complexity**. Spearman correlation heatmaps showing relationships between keystone taxa relative abundance and network complexity metrics across four experimental treatments: Control, Warming, Nitrogen addition and Dry-wet cycling. Upper panels (A-D) show fungal correlations; lower panels (E-H) show bacterial correlations. Each panel displays correlations for total keystone taxa, module hubs, connectors, and dominant phyla (Ascomycota for fungi; multiple phyla for bacteria). Cell colors indicate correlation coefficients (*ρ*): red = positive, blue = negative. Significance levels after FDR correction: **P* < 0.05, ***P* < 0.01, ****P* < 0.001. *n* = 45 samples per treatment.


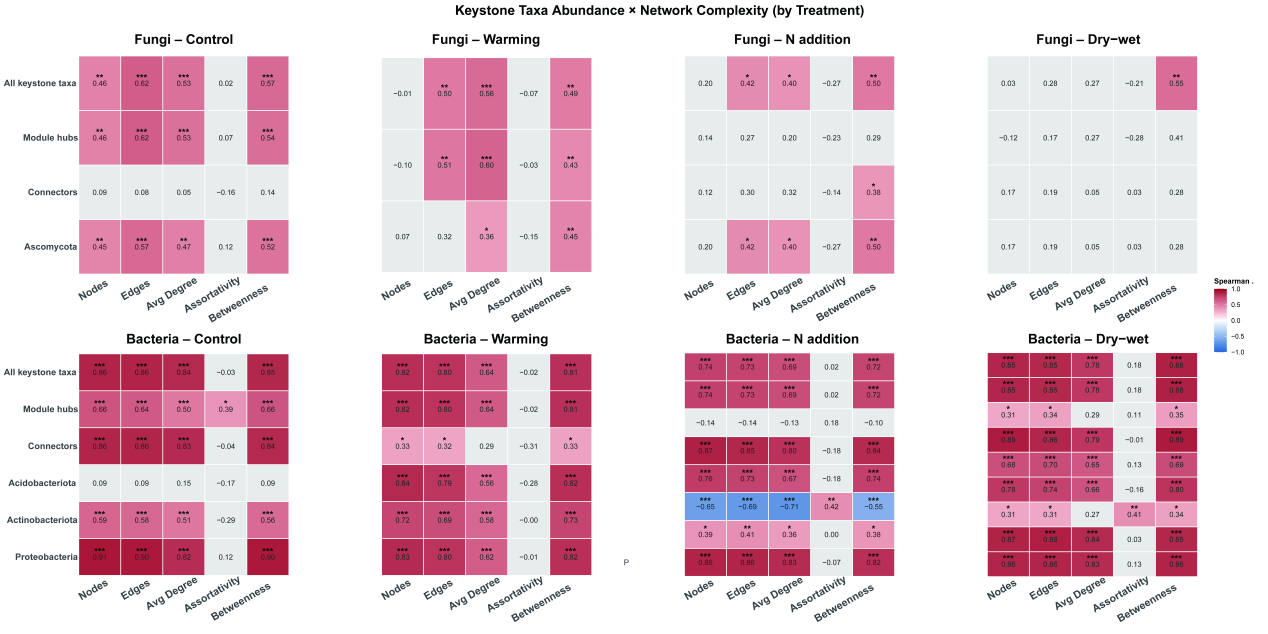

Supplement: Multimedia component 1 [file mmc1.docx]
